# Antibody and Memory B-Cell Immunity in a Heterogeneously SARS-CoV-2-Infected and -Vaccinated Population

**DOI:** 10.1128/mbio.00840-22

**Published:** 2022-06-23

**Authors:** Eva Bednarski, Perla M. Del Rio Estrada, Justin DaSilva, Celia Boukadida, Fengwen Zhang, Yara A. Luna-Villalobos, Ximena Rodríguez-Rangel, Elvira Pitén-Isidro, Edgar Luna-García, Dafne Díaz Rivera, Dulce M. López-Sánchez, Daniela Tapia-Trejo, Maribel Soto-Nava, Myriam Astorga-Castañeda, José O. Martínez-Moreno, Guadalupe S. Urbina-Granados, José A. Jiménez-Jacinto, Francisco J. Serna Alvarado, Yerania E. Enriquez-López, Oliva López-Arellano, Gustavo Reyes-Teran, Paul D. Bieniasz, Santiago Avila-Rios, Theodora Hatziioannou

**Affiliations:** a Laboratory of Retrovirology, The Rockefeller University, New York, New York, USA; b Centro de Investigación en Enfermedades Infecciosas, Instituto Nacional de Enfermedades Respiratorias Ismael Cosío Villegas, Mexico City, Mexico; c Jurisdicción Sanitaria Coyoacán, Servicios de Salud Pública de la Ciudad de México, Mexico City, Mexico; d Jurisdicción Sanitaria Cuajimalpa, Servicios de Salud Pública de la Ciudad de México, Mexico City, Mexico; e Jurisdicción Sanitaria Magdalena Contreras, Servicios de Salud Pública de la Ciudad de México, Mexico City, Mexico; f Jurisdicción Sanitaria Iztapalapa, Servicios de Salud Pública de la Ciudad de México, Mexico City, Mexico; g Jurisdicción Sanitaria Tlalpan, Servicios de Salud Pública de la Ciudad de México, Mexico City, Mexico; h Secretaría de Salud, Mexico City, Mexico; i Institutos Nacionales de Salud y Hospitales de Alta Especialidad, Secretaría de Salud de México, Mexico City, Mexico; j Howard Hughes Medical Institute, Chevy Chase, Maryland, USA; Columbia University College of Physicians & Surgeons

**Keywords:** SARS-CoV-2, neutralizing antibodies, memory B cells, vaccination, infection

## Abstract

Global population immunity to severe acute respiratory syndrome coronavirus 2 (SARS-CoV-2) is accumulating through heterogeneous combinations of infection and vaccination. Vaccine distribution in low- and middle-income countries has been variable and reliant on diverse vaccine platforms. We studied B-cell immunity in Mexico, a middle-income country where five different vaccines have been deployed to populations with high SARS-CoV-2 incidences. Levels of antibodies that bound a stabilized prefusion spike trimer, neutralizing antibody titers, and memory B-cell expansion correlated with each other across vaccine platforms. Nevertheless, the vaccines elicited variable levels of B-cell immunity, and the majority of recipients had undetectable neutralizing activity against the recently emergent omicron variant. SARS-CoV-2 infection, experienced before or after vaccination, potentiated B-cell immune responses and enabled the generation of neutralizing activity against omicron and SARS-CoV for all vaccines in nearly all individuals. These findings suggest that broad population immunity to SARS-CoV-2 will eventually be achieved but by heterogeneous paths.

## INTRODUCTION

The emergence of severe acute respiratory syndrome coronavirus 2 (SARS-CoV-2) and variants thereof has highlighted how viral immune evasion and variable global access to vaccines can profoundly impact the course of pandemics. Thus far, in the coronavirus disease 2019 (COVID-19) pandemic, numerous SARS-CoV-2 vaccines based on a single, nearly ancestral SARS-CoV-2 variant have been developed and differentially deployed around the world (1–5). The immunogenicity and ability of the distinct vaccine platforms to prevent infection and impact the clinical sequelae of SARS-CoV-2 infection have been variable (1–5). Moreover, over time, the ability of all SARS-CoV-2 vaccines to prevent infection has generally deteriorated as immune responses have waned and variants with increased transmissibility and immune evasiveness have displaced prior variants (6–14).

While multiple components of the immune response to SARS-CoV-2 antigens likely contribute to the effectiveness of vaccines in preventing infection and disease, the most definitive correlate of protection against infection is the titer of neutralizing antibodies (15, 16). Moreover, the path that SARS-CoV-2 spike evolution has followed has clearly indicated that neutralizing antibodies have imposed selective pressure on viral populations (17–20). A corollary of this observation is that recently emerging SARS-CoV-2 variants, in particular B.1.1.529 (omicron), have a substantial degree of resistance to neutralizing antibodies elicited by earlier variants and B.1-based vaccines (6, 10–15). The majority of studies on SARS-CoV-2 vaccine-elicited neutralizing antibody responses have focused on single vaccines corresponding to those distributed in high-income countries. However, in low- and middle-income countries, vaccine deployment is far less uniform, and it is therefore important to determine the levels of immunity following the administration of vaccines that represent those deployed globally (21, 22). Such data should help inform policy recommendations regarding additional vaccination doses and nonpharmaceutical interventions to mitigate the disease burden.

Here, we measured antibody and memory B-cell immunity in a sample population comprising 197 vaccinated individuals in Mexico, a middle-income country in which five different vaccines were deployed from 2020 to 2021. We measured spike-binding and neutralizing antibody titers as well as the numbers of spike-specific memory B cells in recipients of each of the five vaccines. We also determined the ability of vaccine recipient plasma to neutralize emergent SARS-CoV-2 variants that have circulated in Mexico, including B.1.1.529 (omicron) as well as an earlier variant, B.1.1.519, originally detected in Mexico (23, 24). Our data reveal significant vaccine-dependent differences in the generation of spike-binding and neutralizing antibodies and the generation of B-cell memory in uninfected and infected vaccine recipients. Nevertheless, these findings also suggest that broad immunity to SARS-CoV-2 variants can be achieved by heterogeneous routes involving various combinations of vaccination and infection.

## RESULTS

### SARS-CoV-2 genomic surveillance and vaccination in Mexico.

**FIG 1:**
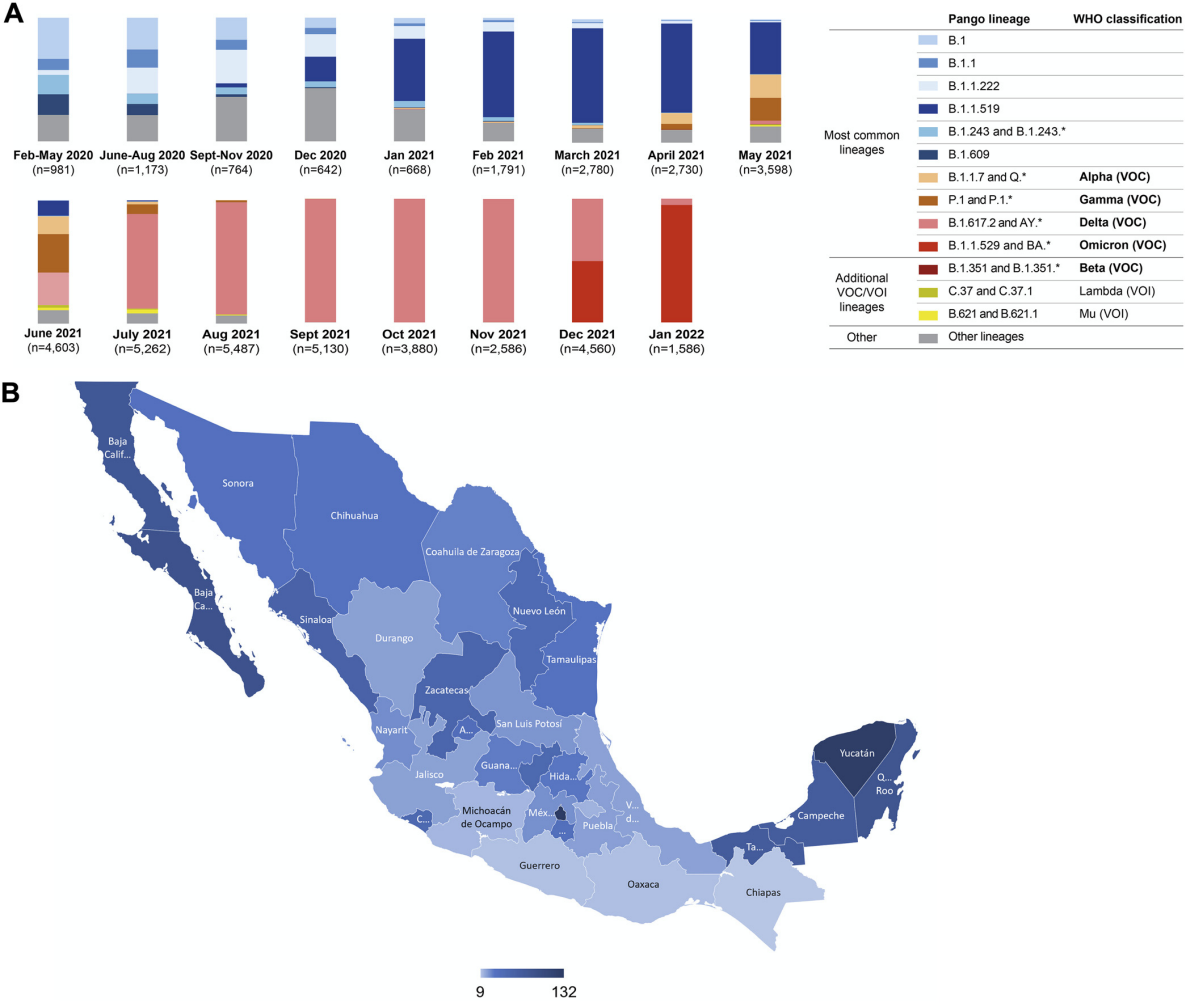
(A) Frequency of SARS-CoV-2 variants in Mexico between February 2020 and January 2022. A total of 48,221 viral genome sequences obtained from samples collected in Mexico and downloaded from the GISAID on 28 January 2022 were analyzed. The frequency of variants was estimated over periods of a few months or individual months based on the numbers of complete genomes sequenced. The most common lineages include variants circulating at frequencies above 10% nationally in at least one period. In addition, less common lineages classified as variants of concern (VOC) and variants of interest (VOI) by the World Health Organization (WHO) were also included. Additional non-VOC/VOI lineages that circulated at frequencies below 10% nationally during all periods were aggregated into the “Other” category. (B) Geographical distribution of viral genomes obtained in Mexico between February 2020 and January 2022. The number of genome sequences per 100,000 persons in the 32 states of Mexico is represented by a color gradient.

Like many countries, Mexico has experienced successive waves of SARS-CoV-2 infection and the dominance of distinct variants over time. To determine the spectrum of SARS-CoV-2 variation that was present in Mexico during the study, we analyzed viral genomes reported between February 2020 and January 2022 to the Global Initiative on Sharing Avian Influenza Data (GISAID) (https://www.gisaid.org/) (Fig. 1A). A total of 48,221 genome sequences were obtained from all 32 states of Mexico, with disparities in regional coverage (Fig. 1B). From February to May 2020, the ancestral B.1 variant and various derivatives thereof dominated but were largely displaced in the winter of 2020 to 2021 by B.1.1.519, a variant that was first described and achieved high prevalence in Mexico but did not spread globally, unlike the contemporary B.1.1.7 (alpha) variant (Fig. 1A). Indeed, the importation of B.1.1.7 (alpha) and P.1 (gamma) was partly responsible for the displacement of B.1.1.519 in the late spring to early summer of 2021, while very few B.1.351 (beta) sequences were also detected during that time. Thereafter, all these variants were displaced by B.1.617 (delta), which was the dominant variant from July to November 2021. As has been the case in numerous other countries, the currently emergent, highly transmissible, and antibody-resistant B.1.1.529 (omicron) variant displaced B.1.617 (delta) during the winter of 2021 to 2022 (Fig. 1A).

From December 2020 to the spring of 2021, five different vaccines were deployed in Mexico, specifically the BNT162b2 mRNA vaccine (Pfizer) (2-dose) as well as the adenovirus-based vaccines ChAdOx1-S (AstraZeneca) (2-dose), Sputnik V (Gamaleya) (2-dose), and Ad5-nCoV (CanSino) (single dose). Finally, an inactivated whole-virion vaccine, CoronaVac (Sinovac) (2-dose) has been used in some locales. To date, these vaccines constitute 98% of the vaccine doses administered in Mexico. We obtained blood samples from 197 individuals within 0.5 to 4.7 months after vaccination. Of note, 80 (40.6%) of the participants had been infected with SARS-CoV-2 prior to the collection of blood samples (see Table S1 in the supplemental material). For 55 participants, infection was documented by a prior positive PCR test in 2020 or 2021, a median of 3.5 months (range, 1 week to 12 months) prior to vaccination (Fig. S1A). While the variant with which these individuals were infected was not determined, the B.1 and B.1.1.159 variants were the most prevalent at the times when PCR diagnoses were made (Fig. 1 and Fig. S1A). A further 25 participants had a positive test result for antibodies against the viral nucleocapsid (N) protein at the time of sampling (mRNA and adenovirus vaccine recipients only), but the time of infection relative to vaccination could not be determined (Table S1). Participants without positive PCR diagnoses and negative anti-N antibody tests were assumed to not have previously been infected. In the case of the CoronaVac recipients, the absence of prior infection could not be unequivocally established due to the presence of vaccine-elicited anti-N antibodies. For this group, participant self-reporting was used to assign prior absence of infection, but it is possible that some CoronaVac recipients had an undiagnosed infection prior to vaccination. The numbers of individuals who received each vaccine were 29 for BNT162b2, 38 for ChAdOx1-S, 57 for Sputnik V, 42 for Ad5-nCoV, and 31 for CoronaVac.

10.1128/mbio.00840-22.1 FIG S1 Participant infection and analysis of NT_50_ values across vaccines. (A) Distribution of participant infection over time. Shown are the numbers of participants who were diagnosed as infected by PCR each month from January 2020 to March 2021. (B) Comparison of neutralizing antibody titers against B.1 across vaccines. NT_50_ values of plasma samples against B.1 from recipients of one of the five SARS-CoV-2 vaccines are indicated. Recipients with no documented prior infection are on the left (closed black circles), and recipients who were infected with SARS-CoV-2 prior to the study as documented by a positive PCR test (closed red circles) or at an unknown time prior to sample collection as indicated by the presence of anti-N antibodies (closed red triangles) are on the right. The median values from 2 to 4 independent experiments for each plasma sample are plotted. Dashed lines indicate the lowest plasma dilution tested (1:50). Lines indicate group median NT_50_ values, which are also indicated in red under each column. Statistical comparisons between each of the vaccines and BNT162b2 were performed using the Mann-Whitney test (GraphPad Prism). (C) Fraction of participants with undetectable neutralizing antibody titers. The percentages of samples for each vaccine group that had NT_50_ values below 50 (the lowest plasma dilution used) against B.1, B1.351 (beta), B.1.617 (delta), and B.1.1.529 (omicron) were calculated for uninfected (−) and infected (+) participants. Download FIG S1, TIF file, 1.3 MB. Copyright © 2022 Bednarski et al. 2022 Bednarski et al. https://creativecommons.org/licenses/by/4.0/ This content is distributed under the terms of the Creative Commons Attribution 4.0 International license.

10.1128/mbio.00840-22.6 TABLE S1 Characteristics of individual participants. Download Table S1, PDF file, 0.1 MB. Copyright © 2022 Bednarski et al. 2022 Bednarski et al. https://creativecommons.org/licenses/by/4.0/ This content is distributed under the terms of the Creative Commons Attribution 4.0 International license.

### Comparison of plasma neutralization potencies and breadths elicited by five SARS-CoV-2 vaccines.

**FIG 2:**
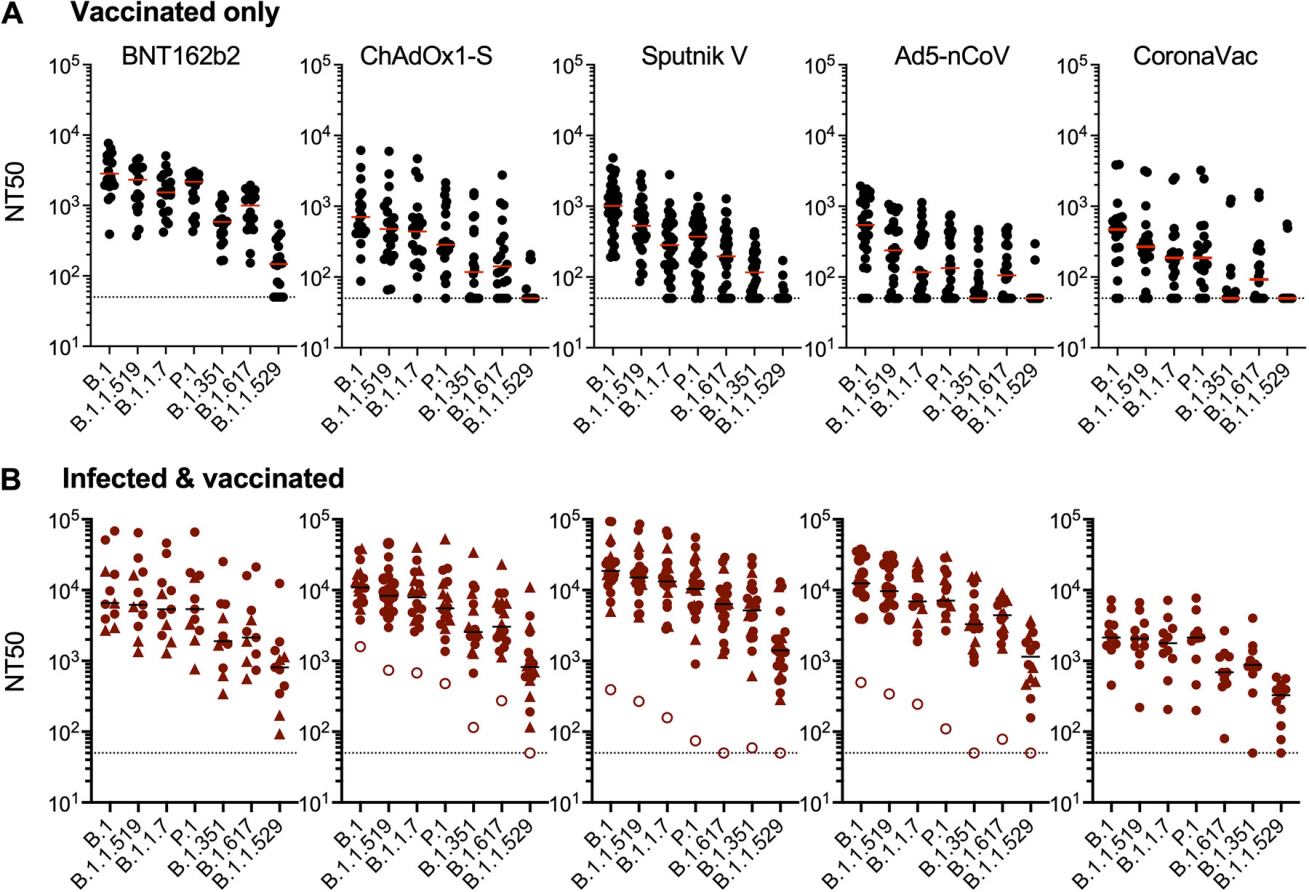
Plasma neutralization activity against SARS-CoV-2 variants in vaccine recipients. NT_50_ values of plasma samples from recipients of one of five SARS-CoV-2 vaccines against B.1 or other SARS-CoV-2 variants were determined. (A) Recipients with no documented prior infection with SARS-CoV-2. (B) Recipients who were infected with SARS-CoV-2 prior to the study as documented by a PCR positive test (closed circles) or at an unknown time prior to sample collection as indicated by the presence of anti-N antibodies (closed triangles). Individuals with prior positive PCR tests but who were seronegative for anti-N are indicated by open circles. The median values from 2 to 4 independent experiments for each plasma sample are plotted. Dashed lines indicate the lowest plasma dilution tested (1:50). Lines indicate group median NT_50_ values.

To compare the abilities of each vaccine to elicit neutralizing antibodies, we employed neutralization assays (25) using pseudoviruses carrying spike proteins derived from the ancestral SARS-CoV-2 variant (B.1) and variants that subsequently emerged in Mexico (Fig. 1). The range of plasma neutralizing titers elicited in previously naive participants varied markedly among the different vaccines (Fig. 2 and Fig. S1B). Specifically, in naive participants, the BNT162b2 mRNA vaccine elicited the highest overall 50% neutralizing titers (NT_50_s) against the ancestral B.1 variant that closely matches the vaccine antigen (median = 2,836) (Fig. S1B). All of the previously uninfected participants who received the 2-dose BNT162b2 vaccine had detectable neutralization titers (range = 390 to 7,651) (Fig. 2A). In contrast, previously uninfected individuals who received the CoronaVac vaccine had the lowest median neutralization titers against B.1 (median NT_50_ = 469 [range = <50 to 3,900]), i.e., 6-fold lower than those of the BNT162b2 vaccine recipients (Fig. 2A and Fig. S1B). For the adenovirus-based vaccines, the single-dose Ad5-nCoV vaccine gave NT_50_ values that were similar to those elicited by CoronaVac (median NT_50_ = 542 [range = <50 to 1,928]), while the 2-dose adenovirus vaccines (ChAdOx1-S and Sputnik V) gave intermediate titers (median NT_50_ = 705 [range = 87 to 6,169] and NT_50_ = 1,013 [range = 190 to 4,815], respectively) (Fig. 2A and Fig. S1B).

For all the deployed vaccines, the neutralization potency of recipient plasma was progressively reduced for variants that subsequently dominated the SARS-CoV-2 viral populations in Mexico. For the B.1.1.519, B.1.1.7 (alpha), and P.1 (gamma) variants, the reduction in potency was comparatively modest: NT_50_ values were 1.2- to 4.6-fold reduced compared to those against B.1 (Fig. 2A). Neutralization potencies against the subsequently emergent, more neutralization-resistant variants were further eroded for all vaccine recipients, with reductions in NT_50_ values ranging from 4.8- to 10.8-fold for B.1.351 (beta) and from 2.8- to 5.1-fold for B.1.617 (delta). Indeed, for several vaccines, NT_50_ values against these variants fell below the limit of detection for a large fraction of participants. This was most noticeable for the CoronaVac vaccine, for which 13/20 and 9/20 plasma samples lacked detectable neutralizing activity against B.1.351 (beta) and B.1.617 (delta), respectively (Fig. S1C).

The recently emergent B.1.1.529 (omicron) variant had the most substantial degree of neutralization resistance. Indeed, the majority (5/17, 16/20, 30/33, 24/26, and 18/20) of plasma samples from uninfected BNT162b2, AstraZeneca, Sputnik, Ad5-nCoV, and CoronaVac recipients, respectively, had undetectable neutralizing activity against B.1.1.529 (omicron) (Fig. 2A and Fig. S1B). Moreover, in the case of the uninfected CoronaVac recipients, these numbers may overestimate the frequency of B.1.1.529 (omicron) neutralizers (see below), as individuals with an undiagnosed prior infection could not be excluded from this group.

### Infection increases neutralizing antibodies for all SARS-CoV-2 vaccines tested.

Previous work has shown that the titers and breadth of neutralizing antibodies elicited following vaccination were increased in individuals who were infected with SARS-CoV-2 months before receiving an mRNA vaccine (18, 26–29). We determined whether this potentiating property of infection in enhancing vaccine-elicited neutralizing antibody titers was generalizable. Of the 197 participants in our cohort, 80 had been infected as well as vaccinated; the majority of these (55 participants) were diagnosed by PCR in 2020 and early 2021, prior to vaccination, while the remaining 25 participants were demonstrated to have anti-N antibodies at the time of sampling (Table S1). Plasma neutralizing titers against the ancestral B.1 variant in these infected and vaccinated individuals compared to the corresponding uninfected groups were increased for all vaccines. Specifically, the median NT_50_ values (ranges) for infected BNT162b2, ChAdOx1-S, Sputnik V, Ad5-nCoV, and CoronaVac recipients were 6,593 (390 to 7,651), 10,893 (1,591 to 38,725), 18,634 (391 to 93,319), 12,462 (494 to 36,770), and 2,133 (454 to 7,259), respectively, representing values that were 2.3-fold to 23-fold higher than those for the uninfected counterpart groups (Fig. 2B). Infection also increased neutralizing antibody titers against the emergent variants compared to those in the uninfected recipients for all vaccines (Fig. 2B). In contrast to uninfected vaccine recipients, nearly all infected and vaccinated individuals had detectable neutralizing activity against the B.1.315 (beta), B.1.617 (delta), and B.1.1.529 (omicron) variants. The median NT_50_ values (ranges) against B.1.1.529 (omicron) were 808 (93 to 12,381), 816 (<50 to 11,102), 1,407 (<50 to 13,025), 1,144 (<50 to 3,742), and 328 (74 to 589) for the infected BNT162b2, ChAdOx1-S, Sputnik V, Ad5-nCoV, and CoronaVac recipients, respectively (Fig. 2B). Notably, 3 out of the 4 infected and vaccinated individuals who lacked detectable neutralizing activity against omicron were negative for anti-N antibodies at the time of sampling (Fig. 2B, open circles), suggesting low or absent prior SARS-CoV-2 antigen exposure, despite a prior positive PCR test. Overall, differences in NT_50_ values between vaccine groups largely dissipated in groups that had been both infected and vaccinated (Fig. S1B).

Demographic characteristics (age and sex) had generally minor, non-statistically significant effects on neutralizing antibody titers. However, the number of individuals in each category was small (Fig. S2A and B). Additionally, in the subset of individuals for whom the time of prior infection documented by a PCR test was available, the time between infection and vaccination did not have a substantial effect on neutralization potency (Fig. S2C).

10.1128/mbio.00840-22.2 FIG S2 Correlation of participant characteristics and neutralizing antibody titers. (A) Participant age in each vaccine group and neutralizing antibody titers (NT_50_). (Top) Recipients with no documented prior infection with SARS-CoV-2 (black circles); (bottom) recipients who were infected with SARS-CoV-2 either prior to the study or at an unknown time prior to or between the times of vaccination and sample collection (red circles). Open circles, participants with prior positive PCR tests but undetectable anti-N antibodies. (B) Participant sex in each uninfected (black) and infected (red) vaccine group and neutralizing antibody titers (NT_50_). *P* values were calculated using 2-way analysis of variance (ANOVA) with Šidák’s multiple-comparison test. (C) Time between infection (positive PCR test) and vaccination and neutralizing antibody titers (NT_50_). Only samples with available PCR test results were included in this analysis. Open circles indicate participants with prior positive PCR tests who were negative for anti-N. Download FIG S2, TIF file, 2.1 MB. Copyright © 2022 Bednarski et al. 2022 Bednarski et al. https://creativecommons.org/licenses/by/4.0/ This content is distributed under the terms of the Creative Commons Attribution 4.0 International license.

### Broad neutralizing activity in infected and heterogeneously vaccinated individuals.

**FIG 3:**
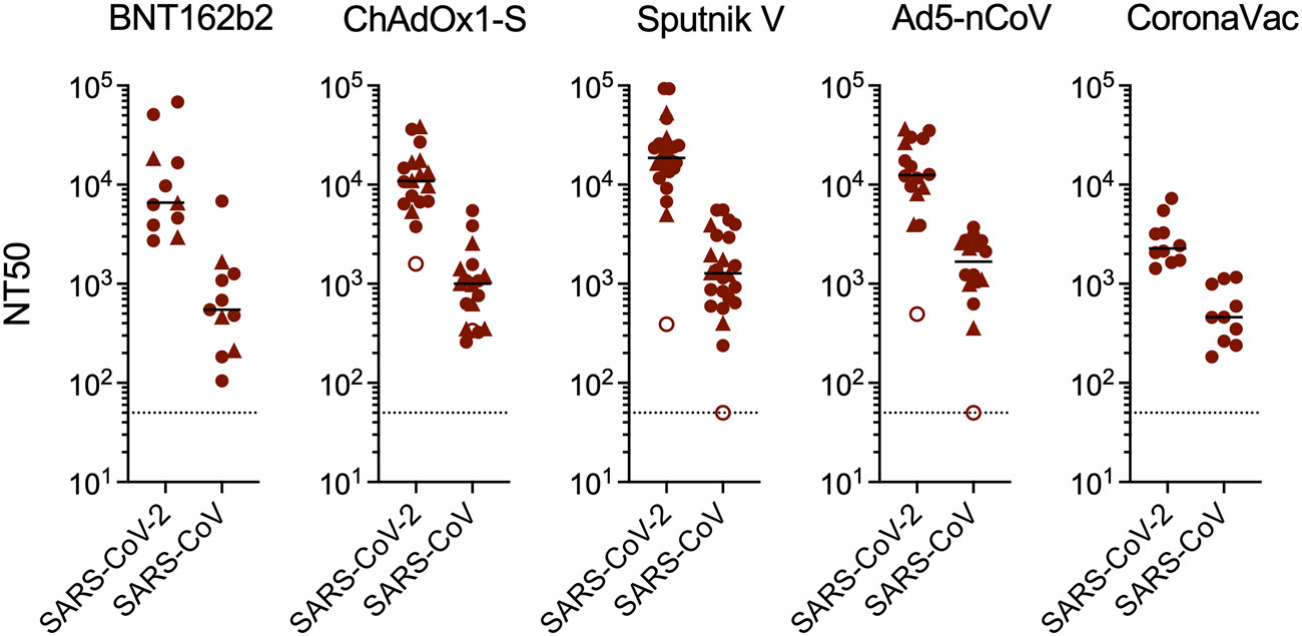
Plasma neutralization activity against SARS-CoV in vaccine recipients. NT_50_ values were measured in recipients who were infected with SARS-CoV-2 either prior to the study as documented by a positive PCR test (closed circles) or at an unknown time prior to sample collection as indicated by the presence of anti-N antibodies (closed triangles). Individuals with prior positive PCR tests but who were seronegative for anti-N are indicated by open circles. The medians from 2 independent experiments for each plasma sample are plotted. Dashed lines indicate the lowest plasma dilution tested (1:50). Lines indicate group median NT_50_ values.

We and others have previously reported that mRNA vaccination of individuals who were previously infected can generate neutralizing activity against distantly related sarbecoviruses such as SARS-CoV (6, 18, 26, 27). To assess the neutralization breadth of plasma samples from infected individuals who received other vaccines, we measured neutralization titers against SARS-CoV (Fig. 3). Nearly all plasma samples from infected vaccine recipients could cross-neutralize SARS-CoV with median NT_50_ values (ranges) of 549 (105 to 6,835), 1,000 (258 to 5,487), 1,276 (238 to 5,575), 1,674 (362 to 3,714), and 460 (183 to 1,162) for the BNT162b2, ChAdOx1-S, Sputnik V, Ad5-nCoV, and CoronaVac recipients, respectively (Fig. 3). Again, the 2 samples lacking SARS-CoV-neutralizing activity were from the individuals who lacked anti-N antibodies while reporting a prior positive PCR test. As expected, titers against SARS-CoV were lower than those against SARS-CoV-2 B.1, but interestingly, the neutralization potency of these plasma samples against SARS-CoV was comparable to that against B.1.1.529 (omicron) despite the far greater divergence of SARS-CoV (Fig. 2B).

### Expansion of spike-specific memory B cells varies among vaccines and correlates with neutralizing titers.

**FIG 4:**
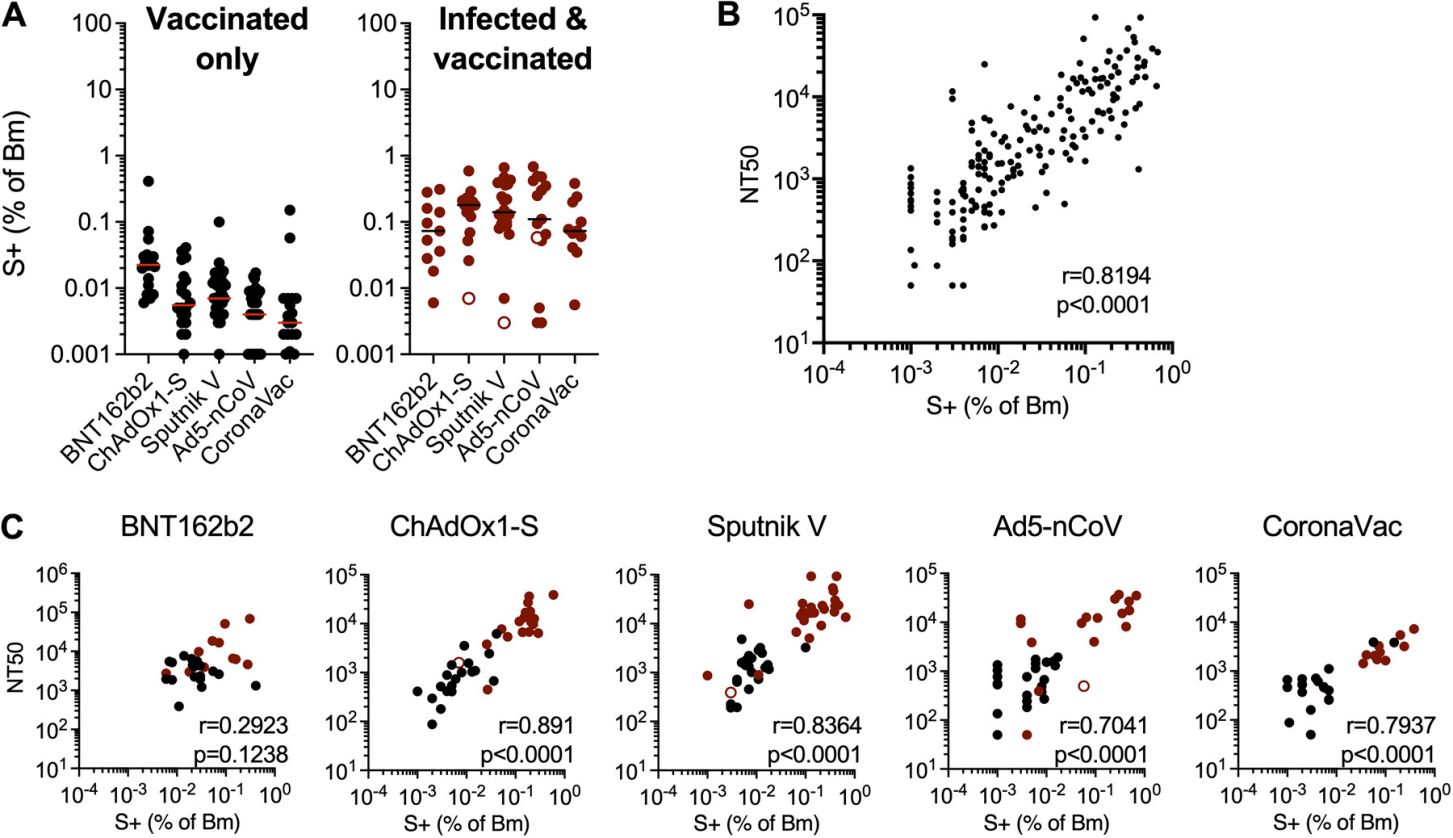
Quantification of SARS-CoV-2 spike-specific memory B cells in vaccine recipients. Memory B cells (Bm) in recipient PBMCs were enumerated using fluorescence-activated cell sorter (FACS) analysis and a trimeric recombinant SARS-CoV-2 (B.1) spike protein. (A) Percentage of spike-binding (S^+^) memory B cells in recipients of one of the five vaccines who were uninfected (left) (black circles) or infected (right) (red circles) (open red circles, anti-N-negative individuals, as described in the legend of Fig. 2B). Horizontal lines indicate the median percentages of S^+^ cells (of memory B cells). (B) Correlation between neutralizing antibody titers (NT_50_) and percentages of S-binding memory B cells across all vaccine recipients. (C) Correlation of neutralizing antibody titers (NT_50_) with percentages of S-binding memory B cells in each separate uninfected (black symbols) or infected (red symbols) vaccine recipient group. The *r* values indicate Spearman correlation coefficients.

While neutralizing antibodies are the only component of the immune response that can, in principle, provide sterilizing protection, anamnestic responses following infection of vaccine recipients may contribute to the prevention of disease. Thus, we measured the percentages of B.1 spike-binding and receptor binding domain (RBD)-binding memory B cells in recipients of each of the five vaccines. In previously uninfected individuals, the percentage of spike-specific memory B cells was highest for BNT162b2 recipients, with a median of 0.022% of all memory B cells being specific for spike, while the lowest median percentage, 0.003%, was found in individuals who received the CoronaVac vaccine (Fig. 4A). The ChAdOx1-S, Sputnik V, and Ad5-nCoV vaccines generated intermediate numbers of spike-specific memory B cells, (medians = 0.006%, 0.007%, and 0.004%, respectively). The numbers of RBD-binding memory B cells in uninfected vaccinated individuals also followed this trend (Fig. S3A).

10.1128/mbio.00840-22.3 FIG S3 Quantification of RBD-specific B cells. RBD-specific memory B cells (Bm) in recipient PBMCs were enumerated using FACS analysis and a recombinant RBD fragment. (A) Percentages of S^+^ specific memory B cells that were specific for the RBD in uninfected (left) (black circles) or infected (right) (red circles) vaccine recipients. Open red circles indicate samples from anti-N-negative individuals as described in the legend of Fig. 2B. Horizontal lines indicate median percentages of RBD^+^ cells (of memory B cells). (B) Correlation of neutralizing antibody titers (NT_50_) with percentages of RBD-binding memory B cells in each vaccine recipient group, colored as described above for panel A. Spearman correlation analysis *r* and *P* values are indicated. Download FIG S3, TIF file, 1.1 MB. Copyright © 2022 Bednarski et al. 2022 Bednarski et al. https://creativecommons.org/licenses/by/4.0/ This content is distributed under the terms of the Creative Commons Attribution 4.0 International license.

Infected and vaccinated individuals had a larger percentage of spike-specific memory B cells than those who were vaccinated but not infected, with median values of 0.073%, 0.18%, 0.14%, 0.11%, and 0.073% for BNT162b2, ChAdOx1-S, Sputnik V, Ad5-nCoV, and CoronaVac recipients, respectively (Fig. 4A). Similarly, the numbers of RBD-binding memory B cells were higher for the infected than for the uninfected vaccine recipients (Fig. S4A). There was, overall, a general equalization of the numbers of memory B cells in infected vaccine recipients compared to those who were vaccinated only, such that the differences between vaccine platforms were ameliorated (Fig. 4B). Notably, the degree to which spike-binding (and RBD-binding) memory B cells were expanded in the blood of vaccine recipients correlated with the plasma neutralization titers in those same individuals (Fig. 4B and C and Fig. S3B). This finding was true for the overall data set (Fig. 4B) and each vaccine (Fig. 4C), except for BNT162b2, where the correlation did not reach statistical significance.

10.1128/mbio.00840-22.4 FIG S4 Correlation of spike-binding antibodies with neutralization of SARS-CoV-2 variants, SARS-CoV, and RBD-specific memory B cells. (A to C) Correlation between spike-binding antibodies (RLU per microliter) and neutralizing antibody titers against B.1.617.2 (delta) (A), B.1.1.519 (omicron) (B), and SARS-CoV (C). (D) Correlation between spike-binding antibodies (RLU per microliter) and percentages of memory B cells that bind the S RBD. Spearman correlation analysis *r* and *P* values are indicated. Download FIG S4, TIF file, 2.1 MB. Copyright © 2022 Bednarski et al. 2022 Bednarski et al. https://creativecommons.org/licenses/by/4.0/ This content is distributed under the terms of the Creative Commons Attribution 4.0 International license.

### Variable levels of antibodies that bind a conformationally intact spike are elicited by different vaccines and predict neutralizing activity and memory B-cell expansion.

**FIG 5:**
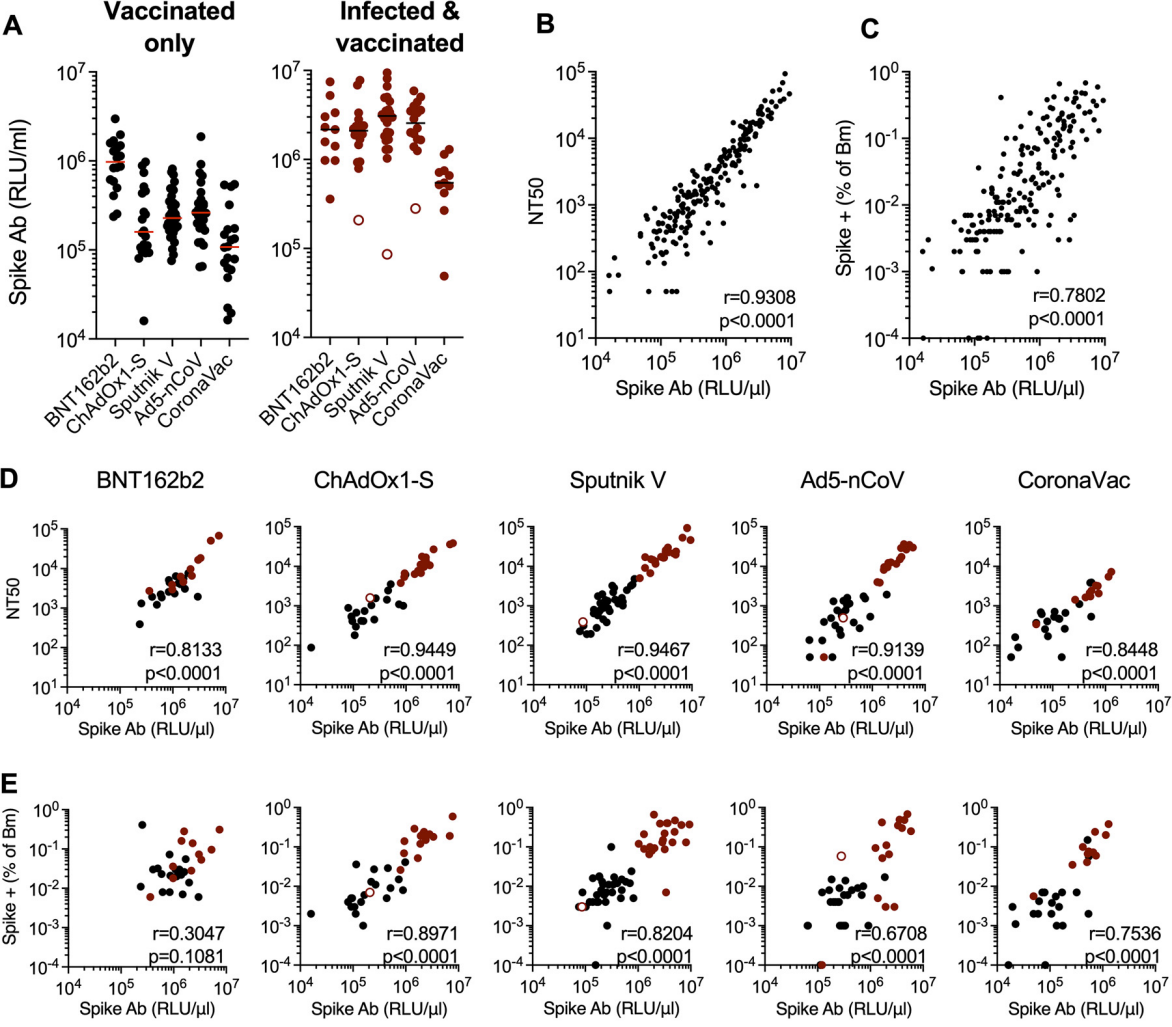
Quantification of antibodies that bind a prefusion SARS-CoV-2 spike protein. A conformationally stabilized trimer of a fusion protein between spike (B.1) and NanoLuc (S-6P-NanoLuc) was used to measure spike-binding antibodies. Antibodies in serially diluted participant plasma samples were captured using protein G magnetic beads and then incubated with S-6P-NanoLuc, and bound NanoLuc activity was quantified. (A) Captured NanoLuc activity expressed as relative light units (RLU) per microliter of plasma from uninfected (left) (black circles) or infected (right) (red circles) vaccine recipients (open red circles indicate anti-N-negative samples, as described in the legend of Fig. 2B). Means from two independent experiments are shown. Lines indicate group median RLU per microliter. (B and C) Correlation between neutralizing antibody (Ab) titers (NT_50_) (B) or spike-specific memory B-cell expansion (C) and spike-binding antibodies (RLU per microliter) across all vaccine recipients. (D and E) Correlation between neutralizing antibody titers (NT_50_) (D) or spike-specific memory B-cell expansion (E) and spike-binding antibodies (RLU per microliter) for each uninfected (black) or infected (red) vaccine recipient group. The *r* values indicate Spearman correlation coefficients.

In addition to neutralizing antibodies, infection or vaccination induces antibodies that bind to spike but do not neutralize. Such antibodies may contribute to the control of SARS-CoV-2 replication *in vivo* (30). To measure spike-binding antibodies, we developed a rapid and convenient assay that specifically measures antibodies that bind to a prefusion spike trimer (19). Specifically, we generated S-6P-NanoLuc, a secreted and conformationally stabilized (HexaPro) form of the SARS-CoV-2 B.1 spike protein with NanoLuciferase luciferase fused at its C terminus. We measured the amount of S-6P-NanoLuc luciferase that bound to participant-derived immunoglobulins captured on protein G magnetic beads. In the uninfected subset of the cohort, the BNT162b2 vaccine elicited the highest spike-binding antibody titers, with median S-6P-NanoLuc-binding titers of 0.97 × 10^6^ relative light units (RLU)/μL of plasma, while the lowest levels of spike-binding antibodies were elicited by CoronaVac (median = 0.1 × 10^6^ RLU/μL) (Fig. 5A). The adenovirus vaccines elicited intermediate levels of spike-binding antibodies (median = 0.16 × 10^6^ to 0.2 × 10^6^ RLU/μL). As was the case for neutralizing antibodies and spike-binding memory B cells, infected and vaccinated participants generated higher levels of spike-binding antibodies (medians = 2.2 × 10^6^, 2.1 × 10^6^, 3.1 × 10^6^, 2.5 × 10^6^, and 0.54 × 10^6^ RLU/μL for BNT162b2, ChAdOx1-S, Sputnik, Ad5-nCoV, and CoronaVac recipients, respectively) (Fig. 5A). The levels of spike-binding antibodies correlated with the neutralizing antibody titers and spike-specific memory B-cell expansion across vaccine platforms (Fig. 5B and C). A correlation between prefusion spike-binding and neutralizing titers was evident for each vaccine tested, and the quantitative relationships between spike-binding antibodies and neutralizing antibodies were similar for each vaccine platform (Fig. 5D). Notably, the levels of antibodies that bound the B.1 spike-based S-6P-NanoLuc also predicted neutralizing activity against B.1.617 (delta), B.1.1.529 (omicron), and SARS-CoV (Fig. S4A to C), even though the quantitative relationship between B.1 spike-binding and neutralizing antibodies against other variants and SARS-CoV was different.

Spike-binding antibody levels also correlated with the numbers of spike- and RBD-specific memory B cells for all vaccines, although the correlation for BNT162b2 did not reach statistical significance (Fig. 5E and Fig. S4D). Overall, therefore, different measurements of B-cell immunity, specifically spike-binding antibodies, neutralizing titers, and spike-specific memory B-cell expansion, correlated with each other across all vaccine platforms tested.

### Longitudinal variation in neutralizing activity.

**FIG 6:**
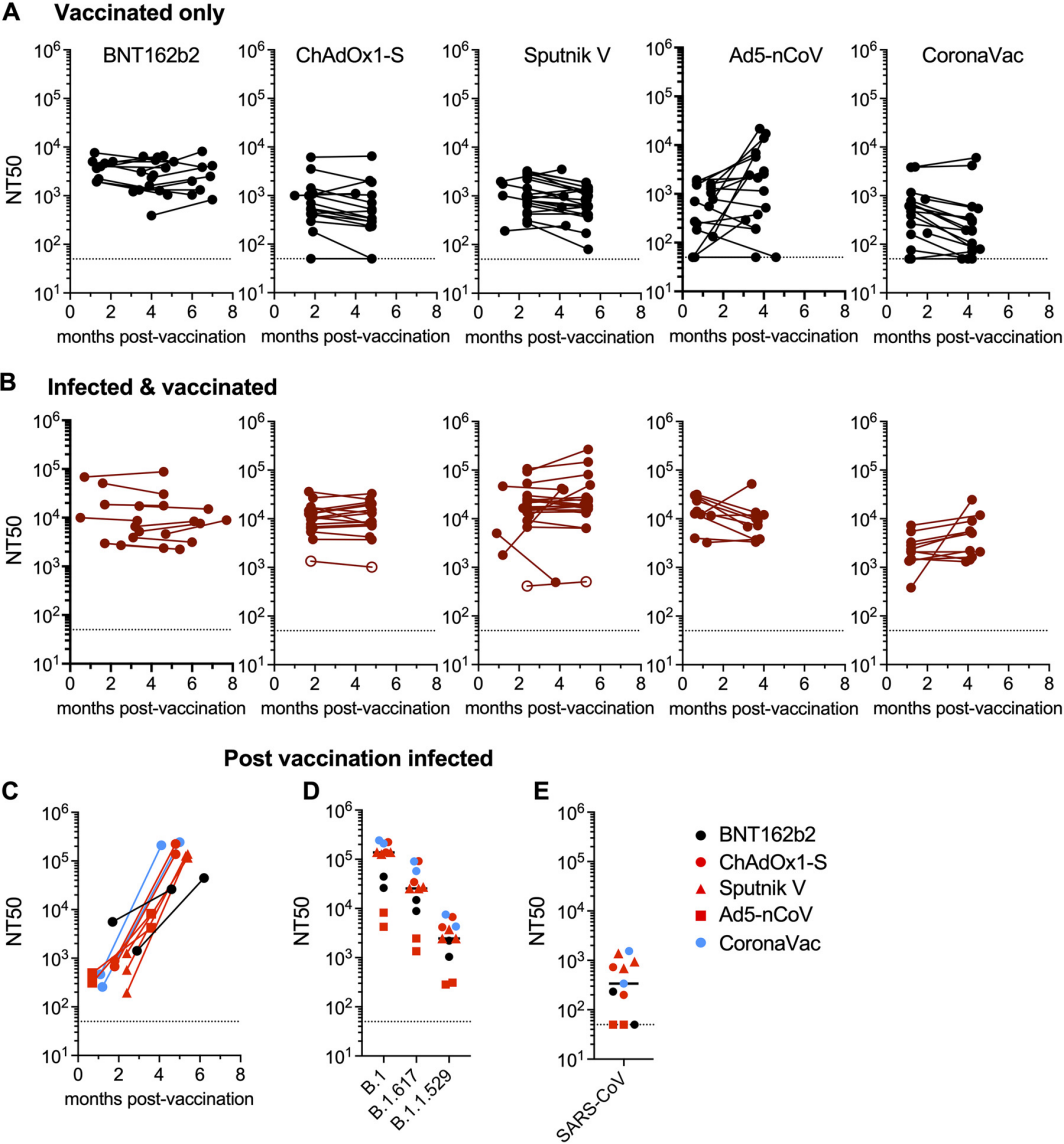
Longitudinal analysis of neutralizing antibodies after vaccination. (A and B) Comparison of neutralizing antibody titers between the first and second plasma samples for each participant. (A) Uninfected participants; (B) SARS-CoV-2-infected participants (open circles, individuals who were seronegative for anti-N, as described in the legend of Fig. 2B). (C) Change in NT_50_ values for participants who were infected between the times of acquisition of the 2 samples as indicated by the acquisition of, or a large increase in, anti-N antibodies. (D and E) Neutralizing antibody titers against SARS-CoV-2 variants (D) and SARS-CoV (E) in the second samples from participants who were infected between the times of collection of the two samples. One sample from the Ad5-nCoV group with a prior positive PCR test and anti-N antibodies in the second but not the first sample was included in this group. The medians from 2 independent experiments for each plasma sample are plotted. Dashed lines indicate the lowest plasma dilution tested (1:50). Horizontal lines indicate group median NT_50_ values.

A caveat associated with the above-mentioned comparisons between vaccine platforms was nonuniformity in the time between receipt of the vaccine and sample collection. We therefore determined the extent to which titers induced in vaccine recipients from all five vaccines used in this study changed over time against B.1 and the two most recently prevalent variants, B.1.617 (delta) and B.1.1.529 (omicron). Most participants (170/197) returned for a follow-up visit 2.5 to 7.8 months after the first (Fig. 6).

For most uninfected participants who remained anti-N negative at both visits, there were small differences in the NT_50_ values measured for B.1 at the second visit compared to the first (Fig. 6A). With the caveat that the timing intervals were not uniform among the vaccine platforms, BNT162b2 vaccine recipients displayed a marginal decrease (~0.9-fold) in neutralizing titers at the second visit, while those in the recipients of ChAdOx1-S, Sputnik V, and CoronaVac increased slightly, ~1.4- to 2.4-fold (Fig. 6A). Ad5-nCoV recipients showed a 4-fold increase in titers against B.1 at the second visit, although this might be due to the first-visit samples being collected very early for the majority of this group (Fig. 6A). The deficit in B.1.617 (delta) and B.1.1.529 (omicron) neutralization at the second visit was similar to that at the first visit for all vaccines (Fig. S5A). As observed for the first visit, NT_50_ values in infected and vaccinated participants were higher than those in the uninfected groups (Fig. 6B and Fig. S5B), and differences in median NT_50_ values between the infected and uninfected groups at the second visit were similar to those at the first. However, in the Ad5-nCoV recipients, the 25-fold difference in NT_50_ values for the infected and uninfected groups at the first visit was only 7-fold at the second visit (Fig. 2A and B and Fig. S5A and B). This discrepancy may be due to the comparatively short interval between vaccination and the first visit for this group, as discussed above.

10.1128/mbio.00840-22.5 FIG S5 Plasma neutralization activity in second-visit samples. NT_50_ values were determined for plasma samples collected from vaccine recipients 2.5 to 7.8 months after the first sample, against B.1 or other variants of concern, as indicated. (A) Recipients who had no documented prior infection with SARS-CoV-2. (B) Recipients who were infected with SARS-CoV-2 either prior to the study as documented by a PCR positive test (closed circles) or at an unknown time prior to or between the times of vaccination and sample collection as confirmed by anti-N tests at the time of sample collection (closed triangles). Open circles, samples from participants with prior positive PCR tests but who had undetectable N titers in the first sample. The medians from 2 independent experiments for each plasma sample are plotted. Dashed lines indicate the lowest plasma dilution tested (1:50). Horizontal lines indicate median NT_50_ values. Download FIG S5, TIF file, 1.1 MB. Copyright © 2022 Bednarski et al. 2022 Bednarski et al. https://creativecommons.org/licenses/by/4.0/ This content is distributed under the terms of the Creative Commons Attribution 4.0 International license.

### Effect of infection after vaccination on neutralizing antibody titers and breadth.

Of the 78 returning mRNA or adenovirus vaccine recipients who were anti-N negative and assumed to not be infected prior to the first sample collection, 8 participants had anti-N antibodies in the second sample (Table S1). A further 2/19 returning recipients of the CoronaVac whole inactivated vaccine exhibited an atypical increase in anti-N titers in the second sample. A single Ad5-nCoV recipient who was reported to be PCR positive but was anti-N negative at the first visit became anti-N positive at the second visit. These 11 participants were judged to be infected during the course of the study, after vaccination, and were considered separately. Based on the times of sample collection, the majority of these postvaccination infections occurred between June and September 2021. B.1.617 (delta) was the most prevalent variant during the majority of this period, but we cannot exclude the possibility that B.1.1.519, B.1.1.7 (alpha), and P.1 (gamma) variants were responsible for postvaccination infections (Fig. 1). In all of these individuals, neutralizing titers increased considerably compared to those at the first visit (Fig. 6C), and the median NT_50_ value (range) was 138,105 (4,227 to 243,267), or 43- to 710-fold higher than those in returning vaccine recipients who did not seroconvert with anti-N antibodies (mRNA and adenovirus vaccine recipients) or did not have increased anti-N titers at the second visit (CoronaVac recipients) (Fig. 6C and D). Plasma samples from all 11 of these postvaccination infected participants were able to neutralize B.1.617 (delta) and B.1.1.529 (omicron), with median NT_50_ values (ranges) of 25,523 (1,349 to 91,522) and 2,457 (284 to 7,511), respectively (Fig. 6D), values comparable to those exhibited by plasma samples from infected and then vaccinated individuals (Fig. 2B and Fig. S5B). Most of the plasma samples from vaccinated and then infected individuals were also able to neutralize SARS-CoV but with potencies that were a median of 7-fold lower than those against B.1.1.529 (omicron) (Fig. 6D and E).

## DISCUSSION

The immune responses elicited by the SARS-CoV-2 mRNA vaccines were extraordinarily effective at providing short-term protection against infection by closely matched SARS-CoV-2 variants (31, 32). Subsequently, waning immunity and the emergence of viral variants with greater transmissibility and antibody evasiveness, including in Mexico (23, 24), have eroded the ability of vaccines to prevent infection (6–14). Most studies have focused on vaccines deployed in wealthier nations, and direct comparisons among vaccines available to less-resource-rich countries are limited (21, 22). Of the five vaccines deployed in Mexico during the period of the study, two, BNT162b2 and ChAdOx1-S, have been the most widely distributed worldwide, in 162 and 182 countries, respectively, while Sputnik V, Ad5-nCoV, and CoronaVac have been used less widely thus far, in 5, 22, and 42 countries, respectively (33). The data presented here reveal important differences in the abilities of vaccines employed in such countries to elicit neutralizing antibodies and B-cell memory in both naive and previously infected individuals and against multiple variants. The ChAdOx1-S, Sputnik V, Ad5-nCoV, and CoronaVac vaccines investigated had variable abilities to elicit neutralizing antibodies, all generating lower titers than those elicited by the BNT162b2 mRNA vaccines against all variants. They are therefore expected to exhibit lower levels of protection against infection. Indeed, many previously uninfected vaccine recipients evaluated here had undetectable levels of neutralizing antibodies against recently emergent SARS-CoV-2 variants. In particular, the majority of previously uninfected vaccine recipients lacked detectable neutralizing activity against omicron and were thus unlikely to be protected against infection.

The deployment of incompletely effective vaccines during an ongoing pandemic has the consequence that population immunity is currently accumulating through heterogeneous routes. In one frequent scenario, infection by SARS-CoV-2 might be followed by the administration of one of a number of vaccines. In another, vaccination might be followed by infection. In a third, an initial vaccination might be followed by boosting with a vaccine of a different type. Finally, in populations with poor vaccination distribution or uptake, immunity might be accrued through multiple infections. In the context of evolving SARS-CoV-2 variants, the nature of exposures to SARS-CoV-2 antigens and levels of immunity are therefore highly variable between individuals. As we and others have previously reported for mRNA vaccines (18, 26–28, 34), prior infection strongly potentiated the ability of each of the vaccines investigated here to elicit neutralizing antibodies. Additionally, prior infection increased the ability of the vaccines to elicit and expand spike- and RBD-specific memory B cells. Prior experience with SARS-CoV-2 antigens and the consequent affinity maturation of B cells (17, 26, 35–37) likely explain why nearly all infected and then vaccinated participants had neutralizing antibodies against omicron and a divergent sarbecovirus, SARS-CoV. A small number of the participants in this study immunized with each of the vaccines acquired infection after vaccination. All of these individuals also exhibited high neutralizing titers, approximately equivalent to those in infected and then vaccinated individuals. Most individuals who were infected after vaccination also had the ability to neutralize omicron and SARS-CoV. Thus, combinations of vaccination and infection might have similar outcomes, irrespective of the order in which they occur. If so, vaccination and infection might progressively build and broaden population immunity and decrease the overall disease burden.

While omicron has spread rapidly among populations with high levels of vaccination, it has nevertheless imposed a reduced disease burden than prior variants. Anamnestic B-cell and T-cell immune responses as well as nonneutralizing and neutralizing antispike antibodies likely contribute to reduced disease severity. Notably, our data demonstrate that a simple quantitative measurement of antibodies that bind to a stabilized prefusion conformation of the spike protein can predict levels of neutralizing antibodies as well as the degrees of spike-specific and RBD-specific memory B-cell expansion. Appropriate serological assays might therefore be useful in the prognostication of the likelihood of mild versus severe outcomes as well as the likelihood of infection in a population. This in turn could inform health care strategies, for example, in a resource-poor setting such as Mexico where multiple different vaccines were utilized, perhaps targeting the deployment of booster shots to those who received vaccines that elicited low levels of antibodies. This information could also guide the use of vaccines that are not widely used in wealthier nations and broaden options when confronting inequity in vaccine distribution, production limitations, as well as hesitancy issues.

## MATERIALS AND METHODS

### Participants.

Over the period from the winter of 2020 to the spring of 2021, the following vaccines became available and were administered to the Mexican population: one based on mRNA, BNT162b2 (Pfizer/BioNTech); three based on adenovirus vector platforms, ChAdOx1-S (AstraZeneca), Sputnik V (Gamaleya), and Ad5-nCoV (CanSino); and one based on whole inactivated virus, CoronaVac (Sinovac). Participants were recruited by the Centre for Research in Infectious Diseases of the National Institute for Respiratory Diseases (INER-CIENI) in Mexico City, in collaboration with municipal authorities of five of the Mayor’s Offices of Mexico City (Coyoacán, Cuajimalpa, Iztapalapa, Magdalena Contreras, and Tlalpan). Each municipality contributed with participants vaccinated with one of the approved vaccines mentioned above, using the recommended doses and intervals, according to the national vaccination plan. All donors provided written informed consent in compliance with protocols set forth and approved by the Comité de Ética en Investigación and the Comité de Investigación (Research Ethics Committee and Research Committee) from the INER Institutional Review Board (study no. B01-21). Blood collection was performed by CIENI-INER staff at health centers of the participating Mayor’s Offices. Epidemiological data of participants were collected on the day of blood donation. Evidence of a positive SARS-CoV-2 test was requested from those participants who declared prior COVID-19 infection before vaccine application. Additionally, SARS-CoV-2 infection was evaluated in all participants by the detection of anti-N antibodies in plasma by an enzyme-linked immunosorbent assay (ELISA) (Elecsys anti-SARS-CoV-2, catalog no. 09203095190; Roche). Participant details are shown in Table S1 in the supplemental material.

Samples were collected between 0.5 and 4.2 months after vaccination from the 197 individuals who had received one of the five different vaccines. Whole blood was collected in acid-citrate-dextrose (ACD) tubes and processed for peripheral blood mononuclear cell (PBMC) and plasma isolation. Plasma samples were obtained after centrifugation and stored at −80°C. PBMCs were obtained from whole blood by Ficoll-Hypaque density gradient centrifugation and cryopreserved at −140°C.

Plasma samples were also collected at a follow-up visit for 170 participants who returned 2.5 to 7.8 months after the first visit. Anti-N titers were measured for all returning participants as described above (Table S1).

### Pseudotyped-virus neutralization assays.

SARS-CoV-2 pseudotyped particles were generated as previously described (25). Briefly, 293T cells were transfected with pNL4-3ΔEnv-nanoluc and pSARS-CoV-2-SΔ19. Forty-eight hours later, particles were harvested, filtered, and stored at −80°C. The amino acid deletions and/or substitutions corresponding to SARS-CoV-2 variants were incorporated into a spike expression plasmid using synthetic gene fragments (IDT) or overlap extension PCR-mediated mutagenesis and Gibson assembly. Specifically, the variant-specific deletions and substitutions introduced into the B1 sequence were as follows: T478K/D614G/P681H/T723A for B.1.1.519, ΔH69/V70/ΔY144/N501Y/A570D/D614G/P681H/T761I/S982A/D1118H for B.1.1.7 (alpha), L18F/T20N/P26S/D138Y/R190S/K417T/E484K/N501Y/D614G/H655Y for P.1 (gamma), D80A/D215G/L242H/R246I/K417N/E484K/N501Y/D614G/A701V for B.1.351 (beta), T19R/Δ156–158/L452R/T478K/D614G/P681R/D950N for B.1.617 (delta), and A76V/Δ69–70/T95I/G214D/Δ143–145/Δ211/L212I/ins214EPE/G339D/S371L/S373P/S375F/K417N/N440K/G446S/S477N/T478K/E484A/Q493K/G498R/N501Y/Y505H/T547K/D614G/H655Y/N679K/P681H/N746K/D796Y/N856K/Q954H/N969K/L981F for B.1.1529 (omicron).

All spike proteins used in the pseudotype neutralization assays had a 19-amino-acid C-terminal deletion and included the R683G substitution, which disrupts the furin cleavage site and increases particle infectivity without grossly affecting antibody sensitivity.

Fivefold serially diluted plasma samples from vaccinated individuals were incubated with SARS-CoV-2 pseudotyped virus for 1 h at 37°C. The mixture was subsequently added to an HT1080-based cell line engineered to express human ACE2 (HT1080.ACE2 cl14). The starting serum dilution on cells was 1:50. NanoLuc luciferase activity in lysates was measured at 48 h postinoculation using the Nano-Glo luciferase assay system (Promega) with Glomax Navigator (Promega). Relative luminescence units were normalized to those derived from cells infected with SARS-CoV-2 pseudotyped virus in the absence of serum. The half-maximal neutralization titers (NT_50_s) for sera were determined using four-parameter nonlinear regression (least-squares regression method without weighting) (constraints, top = 1 and bottom = 0) (GraphPad Prism) and the median values were calculated for each sample.

### Spike-binding antibody assay.

A recombinant conformationally stabilized (HexaPro) and secreted (transmembrane and cytoplasmic domain-deleted) form of the SARS-CoV-2 B.1 spike protein was generated with NanoLuc luciferase, a 3CL protease site, and a 6×His tag fused at its C terminus (S-6P-NanoLuc). The S-6P-NanoLuc protein was expressed in 293-Expi cells and captured on Ni^2+^ magnetic beads, and the purified protein was eluted following incubation with 3CL protease. Plasma samples from vaccinated individuals were initially diluted 1:10 in 2% bovine serum albumin (BSA)–phosphate-buffered saline (PBS) and then serially diluted 5-fold in the wells of a 96-well plate. Immunoglobulins were captured on protein G magnetic beads (Lytic Solutions) diluted in 2% BSA–PBS for 1 h at 4°C. A purified human monoclonal antibody against SARS-CoV-2 spike, C144, diluted 1:20 in 2% BSA–PBS and serially diluted, was used as a positive control. The immunoglobulin-coated beads were washed twice with 2% BSA–PBS and mixed with 10 ng of S-6P-NanoLuc. After a 2-h incubation at 4°C, beads were washed twice and transferred to a fresh 96-well plate. Fifty microliters of reporter lysis buffer (Promega) was added to the beads, and antibody-captured NanoLuc luciferase activity was measured using the Nano-Glo luciferase assay system (Promega) with Glomax Navigator (Promega). Nonparametric Spearman’s correlation analysis (GraphPad Prism) was performed, and *P* values were calculated by a two-tailed analysis with 95% confidence intervals.

### Measurement of spike-binding memory B cells.

Quantification of B cells specific to SARS-CoV-2 S (and RBD) was performed using S (and RBD) proteins multimerized with fluorescently labeled streptavidins based on methods described previously (29). To multimerize SARS-CoV-2 proteins with fluorescently labeled streptavidins, biotinylated SARS-CoV-2 spike protein (catalog no. 793806; BioLegend) was mixed with streptavidin phycoerythrin (PE) (catalog no. 554061; BD) and streptavidin Alexa Fluor 647 (catalog no. 405237; BioLegend) at an ~6:1 molar ratio, and biotinylated RBD protein (catalog no. 793906; BioLegend) was mixed with streptavidin allophycocyanin (APC) Cy7 (catalog no. 554063; BD) at an ~4:1 molar ratio. Biotinylated proteins were incubated with the corresponding streptavidin at 4°C for 1 h. Streptavidin Brilliant Violet 510 (BV510) (catalog no. 405234; BioLegend) was used as a decoy probe, and free biotin from the avidin-biotin blocking system (catalog no. 927301; BioLegend) was used to help avoid cross-reactivity of antigen probes. Cryopreserved cells were thawed and rested overnight in complete medium (RPMI 1640, 10% heat-inactivated fetal bovine serum, 1% penicillin, 1 μg/mL streptomycin, and 1% l-glutamine) prior to staining. Ten million PBMCs were first incubated for 1 h at 4°C with a cocktail containing SARS-CoV-1 multimerized proteins (200 ng of spike protein, 100 ng per probe, and 27.5 ng of RBD protein). Free biotin was added to this cocktail (5 mL). Cells were then washed twice by adding 3 mL of stain buffer (catalog no. 554657; BD) and centrifuged (1,500 rpm for 10 min). Cells were stained with surface antibodies and a viability marker (Aqua dye fluorescent reactive dye, catalog no. L344957; Invitrogen) as follows: cells were incubated with IgG BV786 (20-min incubation at 4°C) (catalog no. 564230, clone G18-145; BD), followed by a 10-min incubation (4°C) with the viability marker and Fc block (catalog no. 422302; BioLegend) and incubation with a cocktail of surface antibodies containing IgM BV605 (clone G20-127, catalog no. 562977; BD), CD19 BV421 (clone HIB19, catalog no. 562440; BD), CD27 peridinin chlorophyll protein (PERCP) Cy5.5 (clone M-T271, catalog no. 560612; BD), IgD PE-Cy7 (clone IA6-2, catalog no. 561314; BD), and CD3 PE-CF594 (clone UCHT1, catalog no. 562280; BD). Cells were washed and fixed with 1% paraformaldehyde diluted in PBS. Stained PBMCs were acquired with a BD FACS Fusion system using FACS DIVA software (v8.0), and data were analyzed using FlowJo v10.8.0 software. The frequency of SARS-CoV-2 S-specific memory B cells was expressed as a percentage of the total memory B cells (analysis strategy of single cells > lymphocytes [forward scatter {FSC} versus side scatter {SSC}] > live cells > CD3-negative cells > CD19-positive cells > CD27-positive cells). The frequency of SARS-CoV-2 RBD-specific memory B cells was measured as a percentage of S-positive (S^+^) memory B cells (gated inside the S-positive cells). Nonparametric Spearman’s correlation analysis (GraphPad Prism) was performed, and *P* values were calculated using a two-tailed test.
