# Supplementary figures and images for: Antibody and Memory B-Cell Immunity in a Heterogeneously SARS-CoV-2-Infected and -Vaccinated Population
Source: mBio. 2022 Jun 23;13(4):e00840-22. doi: 10.1128/mbio.00840-22 (PMC9426429; doi:10.1128/mbio.00840-22)

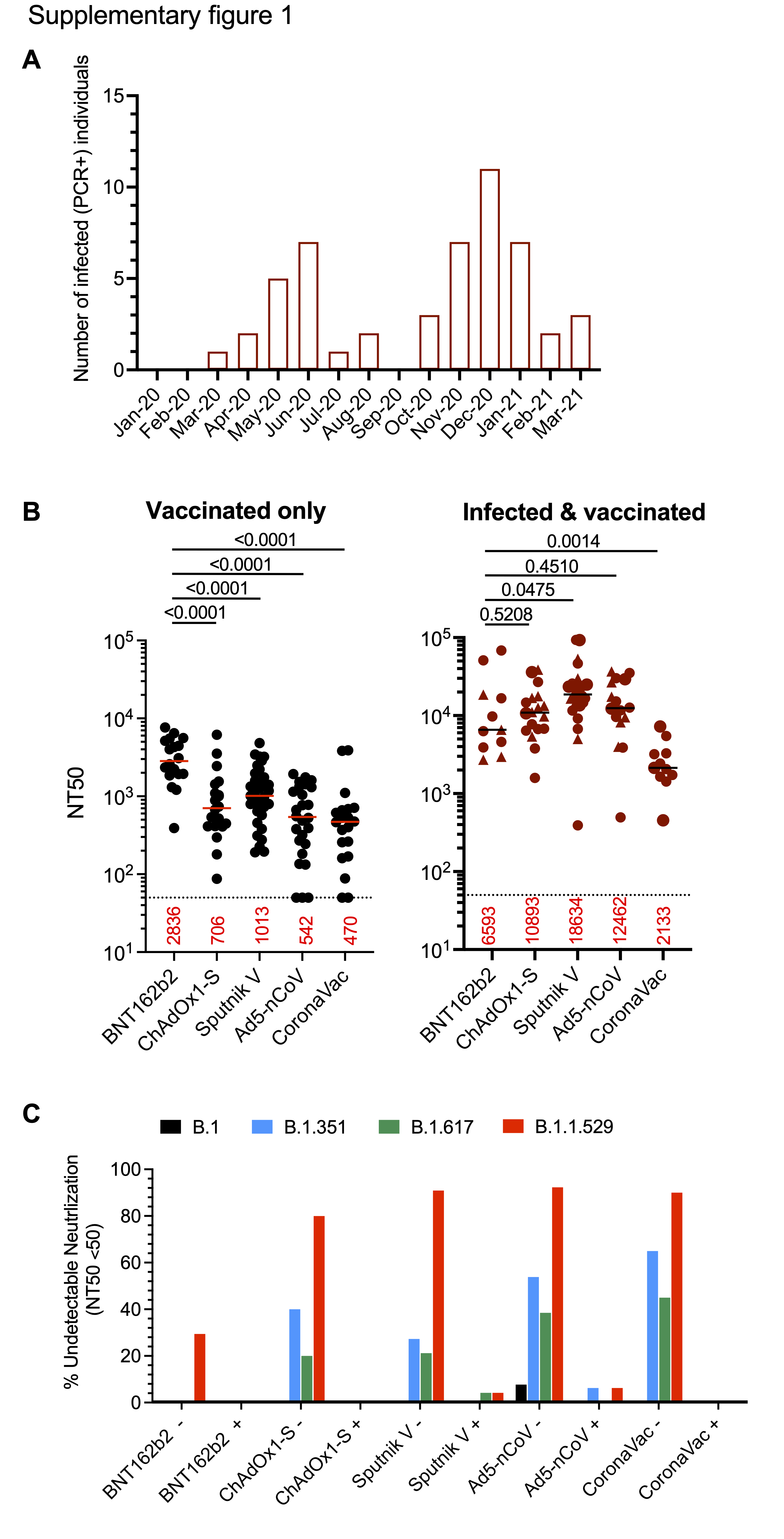

Supplement: FIG S1 [file mbio.00840-22-s0001.tif]

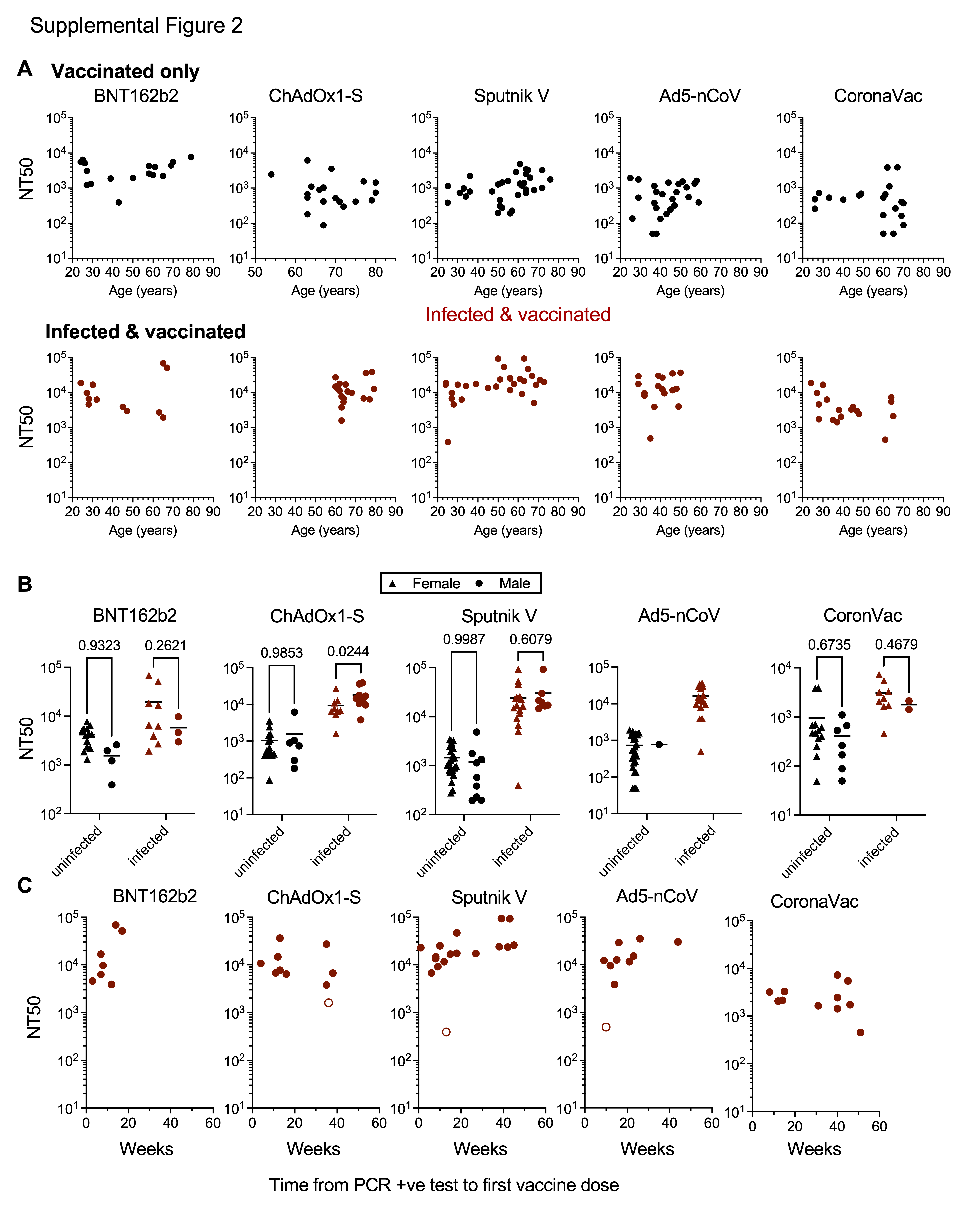

Supplement: FIG S2 [file mbio.00840-22-s0002.tif]

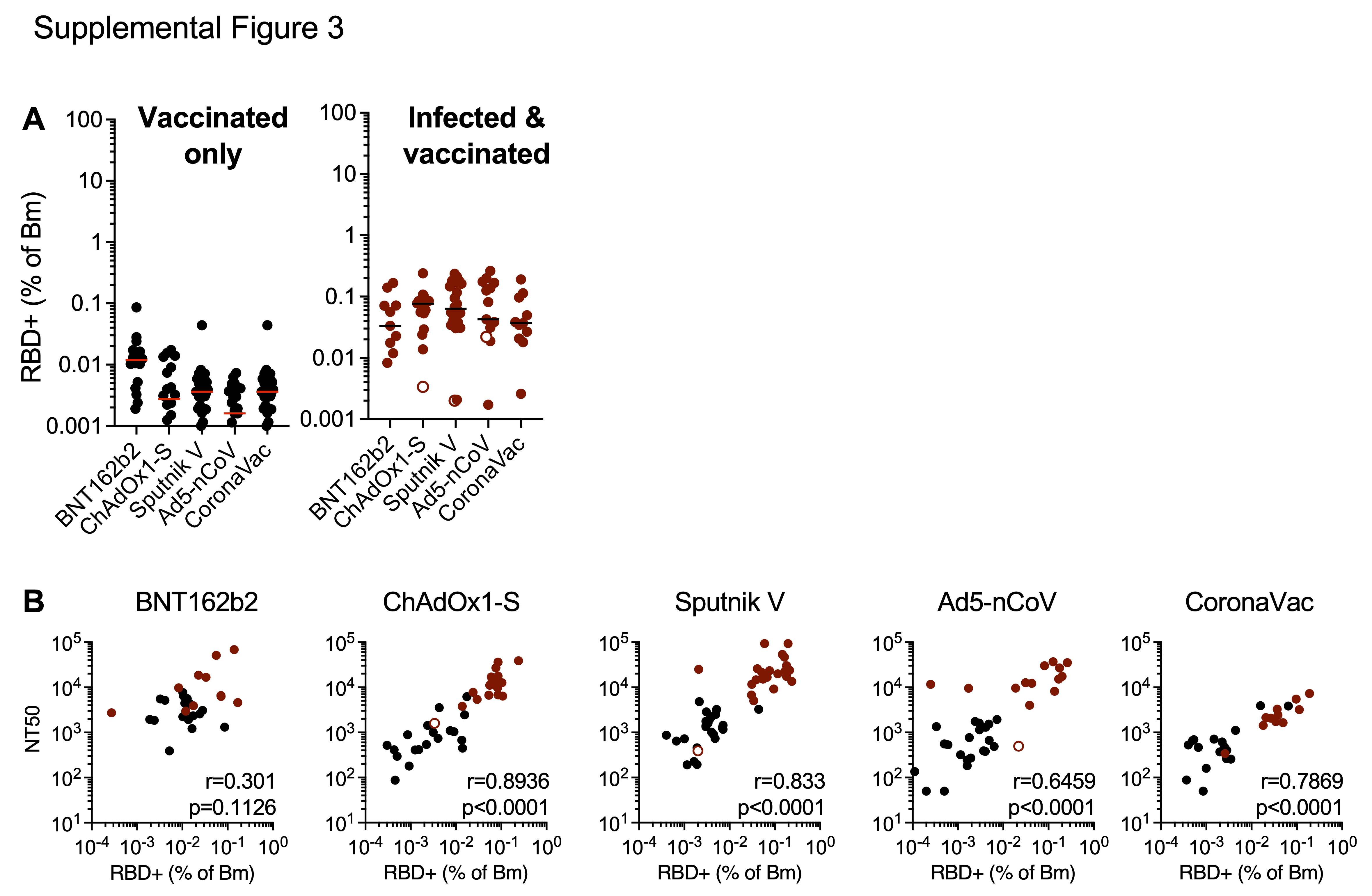

Supplement: FIG S3 [file mbio.00840-22-s0003.tif]

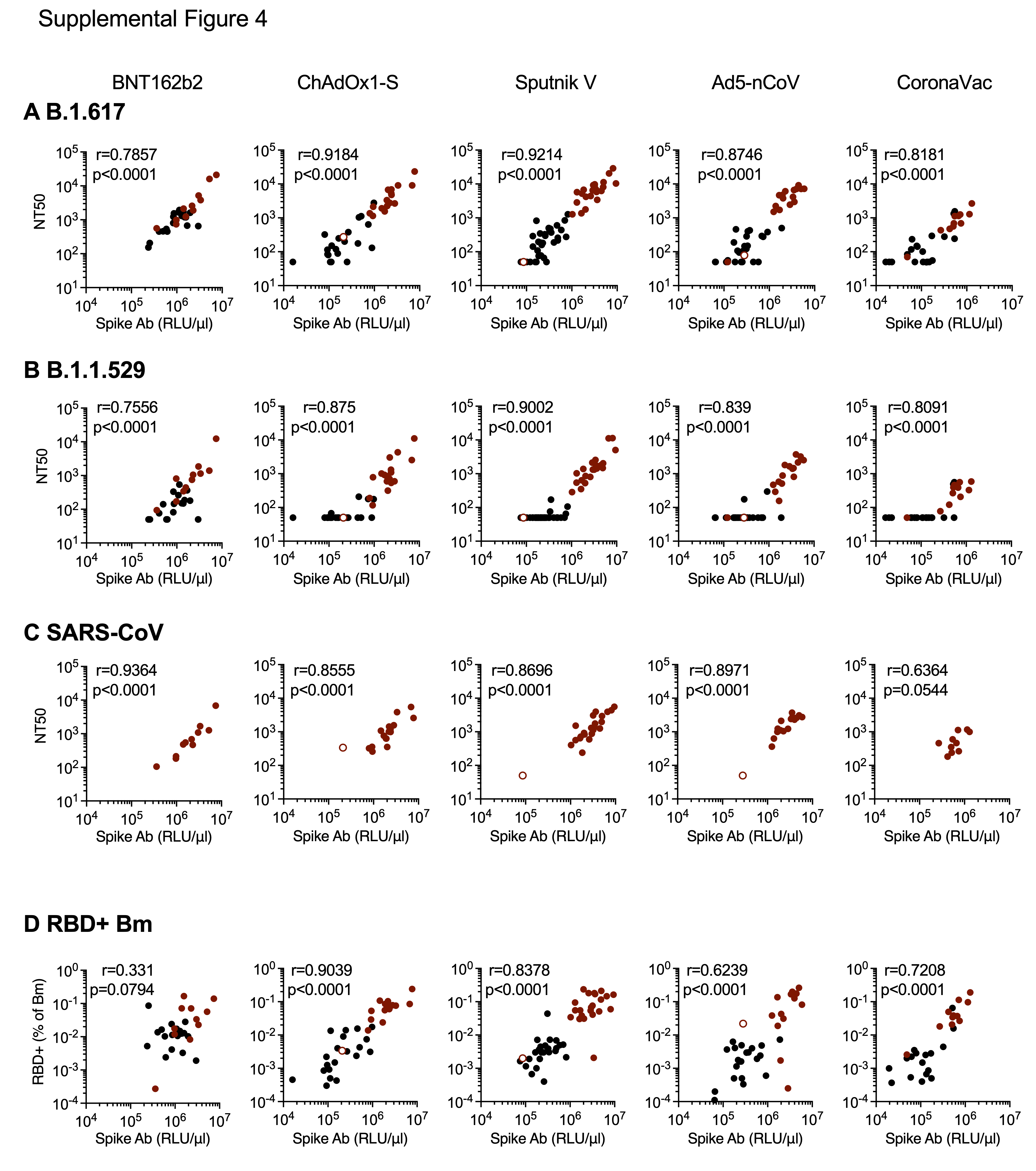

Supplement: FIG S4 [file mbio.00840-22-s0004.tif]

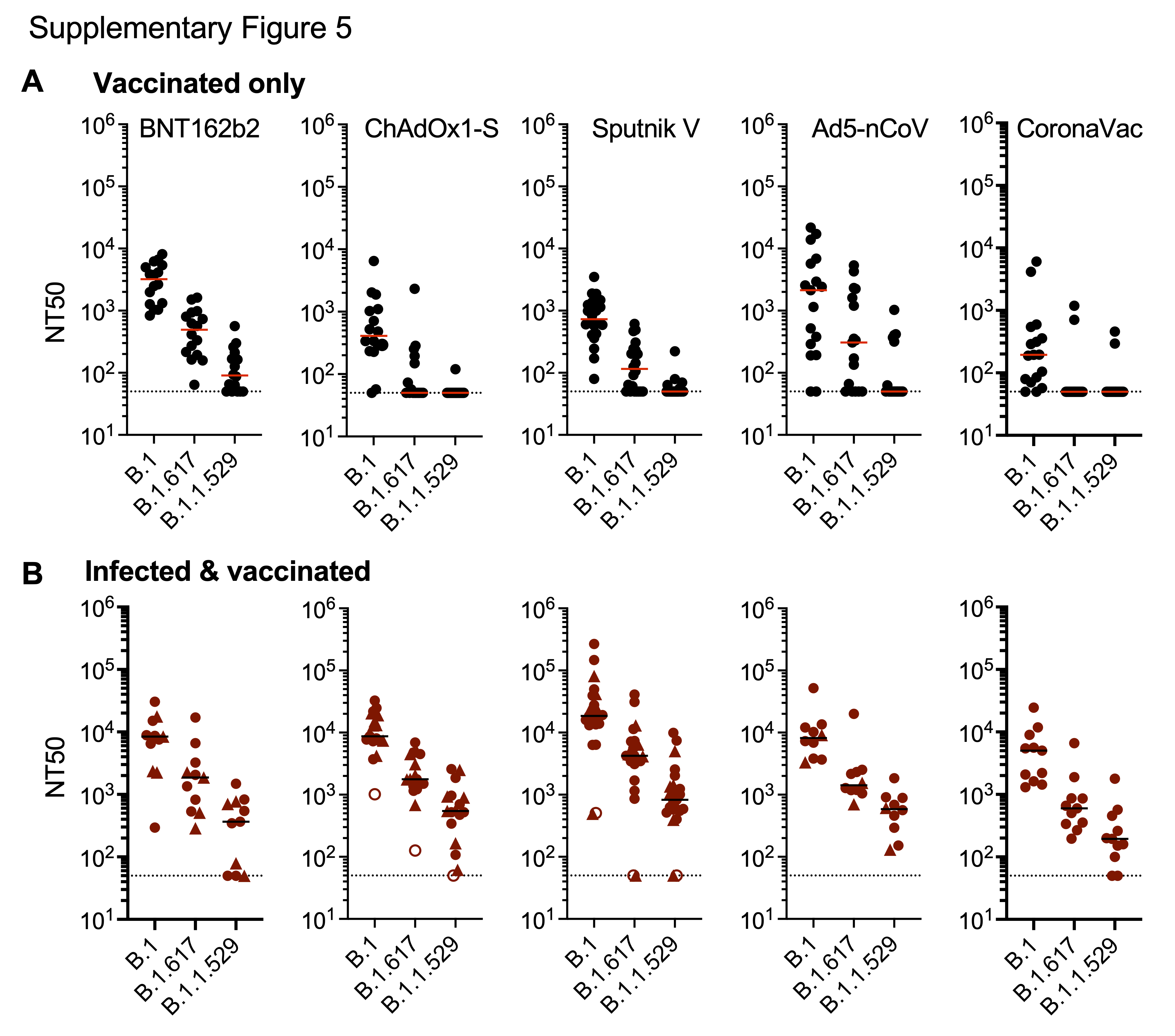

Supplement: FIG S5 [file mbio.00840-22-s0005.tif]
