# Supplementary material for: Antibody and Memory B-Cell Immunity in a Heterogeneously SARS-CoV-2-Infected and -Vaccinated Population
Source: mBio. 2022 Jun 23;13(4):e00840-22. doi: 10.1128/mbio.00840-22 (PMC9426429; doi:10.1128/mbio.00840-22)
Supplement: TABLE S1 [file mbio.00840-22-s0006.pdf]

Table 1. Individual participant characteristics

ID	Age (years)	Sex	Race	Ethnicity	COVID-19 history	Vaccine received		Prior PCR diagnosis	anti-N*	
						1st dose	2nd dose		1st-visit	2nd visit
B01-21/114/GJF	70	F	Caucasian	Hispanic	No	ChAdOx1-S	ChAdOx1-S		negative	negative
B01-21/115/PJF	71	F	Caucasian	Hispanic	No	ChAdOx1-S	ChAdOx1-S		negative	N/A
B01-21/116/JEV	64	F	Caucasian	Hispanic	No	ChAdOx1-S	ChAdOx1-S		negative	negative
B01-21/117/FHG	77	F	Caucasian	Hispanic	No	ChAdOx1-S	ChAdOx1-S		negative	negative
B01-21/119/MdlAPM	63	F	Caucasian	Hispanic	No	ChAdOx1-S	ChAdOx1-S		negative	negative
B01-21/120/JRMM	67	M	Caucasian	Hispanic	No	ChAdOx1-S	ChAdOx1-S		negative	negative
B01-21/121/HGE	64	F	Caucasian	Hispanic	Yes	ChAdOx1-S	ChAdOx1-S		107.3	19.8
B01-21/122/MIRG	67	F	Caucasian	Hispanic	No	ChAdOx1-S	ChAdOx1-S		negative	negative
B01-21/124/AAT	79	F	Caucasian	Hispanic	Yes	ChAdOx1-S	ChAdOx1-S		57.71	25.31
B01-21/125/RDA	61	M	Caucasian	Hispanic	Yes	ChAdOx1-S	ChAdOx1-S		23.96	12.77
B01-21/126/JLMG	66	M	Caucasian	Hispanic	No	ChAdOx1-S	ChAdOx1-S		negative	negative
B01-21/127/AMG	63	F	Caucasian	Hispanic	No	ChAdOx1-S	ChAdOx1-S		negative	98.3
B01-21/128/MRA	75	F	Caucasian	Hispanic	No	ChAdOx1-S	ChAdOx1-S		negative	negative
B01-21/129/EMS	80	F	Caucasian	Hispanic	No	ChAdOx1-S	ChAdOx1-S		negative	negative
B01-21/130/MESS	69	F	Caucasian	Hispanic	No	ChAdOx1-S	ChAdOx1-S		negative	negative
B01-21/131/FRS	80	M	Caucasian	Hispanic	No	ChAdOx1-S	ChAdOx1-S		negative	negative
B01-21/134/JLDIVM	68	M	Caucasian	Hispanic	Yes	ChAdOx1-S	ChAdOx1-S		108.7	74.09
B01-21/135/EM	62	F	Caucasian	Hispanic	Yes	ChAdOx1-S	ChAdOx1-S		19.84	9.83
B01-21/137/MZM	65	M	Caucasian	Hispanic	Yes	ChAdOx1-S	ChAdOx1-S		26.97	14.51
B01-21/139/MELS	67	F	Caucasian	Hispanic	No	ChAdOx1-S	ChAdOx1-S		negative	negative
B01-21/141/RGG	78	M	Caucasian	Hispanic	Yes	ChAdOx1-S	ChAdOx1-S		128.2	negative
B01-21/142/LBOS	63	M	Caucasian	Hispanic	No	ChAdOx1-S	ChAdOx1-S		negative	negative
B01-21/145/ASL	54	F	Caucasian	Hispanic	No	ChAdOx1-S	ChAdOx1-S		negative	negative
B01-21/147/GGLA	67	F	Caucasian	Hispanic	No	ChAdOx1-S	ChAdOx1-S		negative	124.5
B01-21/148/FEV	72	M	Caucasian	Hispanic	No	ChAdOx1-S	ChAdOx1-S		negative	negative
B01-21/149/LMG	63	M	Caucasian	Hispanic	No	ChAdOx1-S	ChAdOx1-S		negative	negative
B01-21/150/CMM	79	F	Caucasian	Hispanic	No	ChAdOx1-S	ChAdOx1-S		negative	negative
B01-21/151/SSG	62	M	Caucasian	Hispanic	Yes	ChAdOx1-S	ChAdOx1-S		15.19	4.61
B01-21/118/MdILMG	63	F	Caucasian	Hispanic	Yes	AztraZeneca	ChAdOx1-S	positive	14.56	7.31
B01-21/123/PHA	74	F	Caucasian	Hispanic	Yes	AztraZeneca	ChAdOx1-S	positive	135.5	66.77
B01-21/132/MVCM	64	F	Caucasian	Hispanic	Yes	AztraZeneca	ChAdOx1-S	positive	2.73	1.24
B01-21/133/PRG	60	M	Caucasian	Hispanic	Yes	AztraZeneca	ChAdOx1-S	positive	100.2	56.91
B01-21/136/LMVM	63	F	Caucasian	Hispanic	Yes	AztraZeneca	ChAdOx1-S	positive	negative	negative
B01-21/138/MLEH	60	F	Caucasian	Hispanic	Yes	AztraZeneca	ChAdOx1-S	positive	27.03	13.78
B01-21/140/JEH	63	M	Caucasian	Hispanic	Yes	AztraZeneca	ChAdOx1-S	positive	19.01	14.46
B01-21/143/SR	77	F	Caucasian	Hispanic	Yes	AztraZeneca	ChAdOx1-S	positive	56.36	23.66
B01-21/144/AM	66	M	Caucasian	Hispanic	Yes	AztraZeneca	ChAdOx1-S	positive	59.79	31.39
B01-21/146/DSYH	75	M	Caucasian	Hispanic	Yes	AztraZeneca	ChAdOx1-S	positive	116.7	negative
B01-21/046/GGER	36	F	Caucasian	Hispanic	No	Ad5-nCoV			negative	negative

**B01-21/068/DMA**	38	F	Caucasian	Hispanic	No	Ad5-nCoV			negative	negative
**B01-21/188/MIMM**	45	F	Caucasian	Hispanic	No	Ad5-nCoV			negative	negative
**B01-21/189/ADS**	41	F	Caucasian	Hispanic	Yes	Ad5-nCoV			61.39	34.17
**B01-21/190/YMCM**	39	F	Caucasian	Hispanic	Yes	Ad5-nCoV		positive	72.29	27.54
**B01-21/191/ALM**	29	F	Caucasian	Hispanic	No	Ad5-nCoV			negative	negative
**B01-21/192/LDOC**	41	F	Caucasian	Hispanic	No	Ad5-nCoV			negative	negative
**B01-21/193/MGM**	29	F	Caucasian	Hispanic	Yes	Ad5-nCoV			13.32	N/A
**B01-21/194/TGG**	32	F	Caucasian	Hispanic	Yes	Ad5-nCoV		positive	34.22	N/A
**B01-21/195/MMNP**	51	F	Caucasian	Hispanic	No	Ad5-nCoV			negative	negative
**B01-21/196/MdPGN**	43	F	Caucasian	Hispanic	No	Ad5-nCoV			negative	negative
**B01-21/197/AVG**	37	F	Caucasian	Hispanic	No	Ad5-nCoV			negative	N/A
**B01-21/198/EMB**	47	F	Caucasian	Hispanic	No	Ad5-nCoV			negative	19.96
**B01-21/199/RFM**	32	F	Caucasian	Hispanic	Yes	Ad5-nCoV			17.39	N/A
**B01-21/200/SMS**	42	F	Caucasian	Hispanic	Yes	Ad5-nCoV			55.82	N/A
**B01-21/201/XHH**	35	F	Caucasian	Hispanic	Yes	Ad5-nCoV		positive	negative	15.95
**B01-21/202/VNA**	48	F	Caucasian	Hispanic	Yes	Ad5-nCoV		positive	43.72	20.78
**B01-21/203/VMM**	48	F	Caucasian	Hispanic	No	Ad5-nCoV			negative	60.25
**B01-21/204/PMC**	46	F	Caucasian	Hispanic	Yes	Ad5-nCoV		positive	26.23	10.96
**B01-21/205/ARV**	38	F	Caucasian	Hispanic	No	Ad5-nCoV			negative	negative
**B01-21/206/HGRB**	39	F	Caucasian	Hispanic	Yes	Ad5-nCoV		positive	61.06	33.29
**B01-21/207/AHA**	46	F	Caucasian	Hispanic	No	Ad5-nCoV			negative	N/A
**B01-21/208/GOO**	38	F	Caucasian	Hispanic	No	Ad5-nCoV			negative	negative
**B01-21/209/LKBC**	29	F	Caucasian	Hispanic	No	Ad5-nCoV			nd	N/A
**B01-21/210/EFSA**	49	F	Caucasian	Hispanic	Yes	Ad5-nCoV			27	4.14
**B01-21/211/EGR**	48	F	Caucasian	Hispanic	No	Ad5-nCoV			negative	N/A
**B01-21/212/SCC**	37	F	Caucasian	Hispanic	No	Ad5-nCoV			negative	N/A
**B01-21/213/AVGL**	25	F	Caucasian	Hispanic	No	Ad5-nCoV			negative	nd
**B01-21/214/AKRO**	26	F	Caucasian	Hispanic	No	Ad5-nCoV			negative	N/A
**B01-21/215/MRS**	49	F	Caucasian	Hispanic	No	Ad5-nCoV			negative	N/A
**B01-21/216/MMVG**	59	F	Caucasian	Hispanic	No	Ad5-nCoV			negative	N/A
**B01-21/217/GGT**	50	F	Caucasian	Hispanic	Yes	Ad5-nCoV			9.71	N/A
**B01-21/273/MCAR**	40	F	Caucasian	Hispanic	No	Ad5-nCoV			negative	negative
**B01-21/274/BHB**	57	F	Caucasian	Hispanic	No	Ad5-nCoV			negative	negative
**B01-21/275/BAdLC**	46	F	Caucasian	Hispanic	Yes	Ad5-nCoV		positive	166.2	139.9
**B01-21/276/CNMA**	37	F	Caucasian	Hispanic	Yes	Ad5-nCoV		positive	53.33	24.8
**B01-21/278/JMBG**	38	M	Caucasian	Hispanic	No	Ad5-nCoV			negative	negative
**B01-21/279/BGPdL**	53	F	Caucasian	Hispanic	No	Ad5-nCoV			negative	negative
**B01-21/280/RTL**	54	F	Caucasian	Hispanic	No	Ad5-nCoV			negative	negative
**B01-21/281/MRA**	58	F	Caucasian	Hispanic	No	Ad5-nCoV			negative	negative
**B01-21/282/AEEH**	44	F	Caucasian	Hispanic	No	Ad5-nCoV			negative	negative
**B01-21/219/AHC**	33	F	Caucasian	Hispanic	No	CoronaVac	CoronaVac		3.39	negative
**B01-21/220/SLR**	60	M	Caucasian	Hispanic	No	CoronaVac	CoronaVac		7.26	1.1

B01-21/221/MFT	39	F	Caucasian	Hispanic	Yes	CoronaVac	CoronaVac	positive	108.6	35.42
B01-21/222/PAR	28	F	Caucasian	Hispanic	No	CoronaVac	CoronaVac		32.33	4.49
B01-21/223/RMV	63	M	Caucasian	Hispanic	No	CoronaVac	CoronaVac		negative	negative
B01-21/224/MBMJ	28	F	Caucasian	Hispanic	Yes	CoronaVac	CoronaVac	positive	75.22	27.99
B01-21/225/NMT	66	M	Caucasian	Hispanic	No	CoronaVac	CoronaVac		negative	negative
B01-21/226/RTG	38	F	Caucasian	Hispanic	Yes	CoronaVac	CoronaVac	positive	82.19	21.8
B01-21/227/AGG	35	F	Caucasian	Hispanic	Yes	CoronaVac	CoronaVac	positive	123.9	31.9
B01-21/228/KHO	26	F	Caucasian	Hispanic	No	CoronaVac	CoronaVac		10.35	negative
B01-21/229/EFT	44	F	Caucasian	Hispanic	Yes	CoronaVac	CoronaVac	positive	21.45	10.9
B01-21/230/RCGG	37	M	Caucasian	Hispanic	Yes	CoronaVac	CoronaVac	positive	111.4	35.77
B01-21/231/CIVA	26	F	Caucasian	Hispanic	No	CoronaVac	CoronaVac		4.03	80.87
B01-21/232/LGLJ	64	F	Caucasian	Hispanic	Yes	CoronaVac	CoronaVac	positive	86.49	52.54
B01-21/233/MGG	48	F	Caucasian	Hispanic	Yes	CoronaVac	CoronaVac	positive	191	107.6
B01-21/234/RGL	70	F	Caucasian	Hispanic	No	CoronaVac	CoronaVac		3.01	N/A
B01-21/235/MFB	61	F	Caucasian	Hispanic	Yes	CoronaVac	CoronaVac	positive	negative	122.2
B01-21/236/MHHD	49	F	Caucasian	Hispanic	No	CoronaVac	CoronaVac		2.64	1.27
B01-21/237/CEPA	48	F	Caucasian	Hispanic	No	CoronaVac	CoronaVac		18.43	2.55
B01-21/238/FAVO	40	F	Caucasian	Hispanic	No	CoronaVac	CoronaVac		6.19	114.3
B01-21/239/MdCDA	69	F	Caucasian	Hispanic	No	CoronaVac	CoronaVac		negative	negative
B01-21/240/ARRM	65	M	Caucasian	Hispanic	Yes	CoronaVac	CoronaVac	positive	101.1	128.7
B01-21/241/SRV	67	F	Caucasian	Hispanic	No	CoronaVac	CoronaVac		173.6	149.2
B01-21/243/HGMDL	62	F	Caucasian	Hispanic	No	CoronaVac	CoronaVac		97.58	26.91
B01-21/245/LSD	64	F	Caucasian	Hispanic	Yes	CoronaVac	CoronaVac	positive	118.6	52.1
B01-21/246/EEGE	65	F	Caucasian	Hispanic	No	CoronaVac	CoronaVac		negative	negative
B01-21/250/MSLH	69	F	Caucasian	Hispanic	No	CoronaVac	CoronaVac		1.69	negative
B01-21/251/JMM	70	M	Caucasian	Hispanic	No	CoronaVac	CoronaVac		negative	negative
B01-21/252/JCHA	60	M	Caucasian	Hispanic	No	CoronaVac	CoronaVac		negative	negative
B01-21/277/JSNF	61	M	Caucasian	Hispanic	No	CoronaVac	CoronaVac		2.54	1.9
B01-21/283/CRCM	60	M	Caucasian	Hispanic	No	CoronaVac	CoronaVac		negative	negative
B01-21/071/LRH	64	F	Caucasian	Hispanic	No	Sputnik V	Sputnik V		negative	negative
B01-21/072/JYGL	24	M	Caucasian	Hispanic	Yes	Sputnik V	Sputnik V	positive	181.9	67.58
B01-21/073/AUG	25	M	Caucasian	Hispanic	No	Sputnik V	Sputnik V		negative	negative
B01-21/074/DSR	25	M	Caucasian	Hispanic	No	Sputnik V	Sputnik V		negative	negative
B01-21/075/KAKM	25	F	Caucasian	Hispanic	Yes	Sputnik V	Sputnik V	positive	negative	negative
B01-21/076/JJMA	49	M	Caucasian	Hispanic	Yes	Sputnik V	Sputnik V	positive	29.71	13.39
B01-21/078/ELMC	47	F	Caucasian	Hispanic	No	Sputnik V	Sputnik V		negative	negative
B01-21/080/GAH	51	F	Caucasian	Hispanic	No	Sputnik V	Sputnik V		negative	negative
B01-21/083/LMMV	36	F	Caucasian	Hispanic	No	Sputnik V	Sputnik V		negative	negative
B01-21/084/NJCA	33	F	Caucasian	Hispanic	No	Sputnik V	Sputnik V		negative	negative
B01-21/085/VMQS	34	M	Caucasian	Hispanic	No	Sputnik V	Sputnik V		negative	17.18
B01-21/088/BACN	34	F	Caucasian	Hispanic	Yes	Sputnik V	Sputnik V		25.2	17.69
B01-21/089/MdCRC	31	F	Caucasian	Hispanic	No	Sputnik V	Sputnik V		negative	N/A

**B01-21/090/CASC**	39	M	Caucasian	Hispanic	Yes	Sputnik V	Sputnik V	positive	65.7	24.88
**B01-21/093/MGMG**	62	F	Caucasian	Hispanic	Yes	Sputnik V	Sputnik V	positive	128.5	81.79
**B01-21/094/MdRAB**	66	F	Caucasian	Hispanic	No	Sputnik V	Sputnik V		negative	negative
**B01-21/095/MdRRR**	61	F	Caucasian	Hispanic	Yes	Sputnik V	Sputnik V	positive	48.17	34.17
**B01-21/096/DCTM**	36	F	Caucasian	Hispanic	No	Sputnik V	Sputnik V		negative	negative
**B01-21/099/VPM**	27	F	Caucasian	Hispanic	Yes	Sputnik V	Sputnik V	positive	150.5	32.23
**B01-21/104/MDOD**	56	F	Caucasian	Hispanic	Yes	Sputnik V	Sputnik V	positive	67.51	31.51
**B01-21/106/MdSLRO**	68	F	Caucasian	Hispanic	Yes	Sputnik V	Sputnik V		35.47	negative
**B01-21/113/AOC**	45	F	Caucasian	Hispanic	Yes	Sputnik V	Sputnik V	positive	52.16	39.56
**B01-21/152/MdPAO**	59	F	Caucasian	Hispanic	No	Sputnik V	Sputnik V		negative	negative
**B01-21/153/MRLC**	76	M	Caucasian	Hispanic	No	Sputnik V	Sputnik V		negative	negative
**B01-21/154/MdICYSO**	72	F	Caucasian	Hispanic	No	Sputnik V	Sputnik V		negative	negative
**B01-21/155/AGA**	56	F	Caucasian	Hispanic	Yes	Sputnik V	Sputnik V	positive	25.66	15.13
**B01-21/157/ACBL**	62	F	Caucasian	Hispanic	No	Sputnik V	Sputnik V		negative	negative
**B01-21/158/LRZ**	68	F	Caucasian	Hispanic	No	Sputnik V	Sputnik V		negative	N/A
**B01-21/159/BLPS**	52	F	Caucasian	Hispanic	No	Sputnik V	Sputnik V		negative	negative
**B01-21/160/BJCV**	65	F	Caucasian	Hispanic	No	Sputnik V	Sputnik V		negative	negative
**B01-21/161/AFO**	60	F	Caucasian	Hispanic	No	Sputnik V	Sputnik V		negative	negative
**B01-21/162/CFRG**	50	M	Caucasian	Hispanic	No	Sputnik V	Sputnik V		negative	45.71
**B01-21/163/FJGD**	56	M	Caucasian	Hispanic	No	Sputnik V	Sputnik V		negative	negative
**B01-21/164/GMGC**	61	M	Caucasian	Hispanic	No	Sputnik V	Sputnik V		negative	N/A
**B01-21/165/EFG**	72	F	Caucasian	Hispanic	No	Sputnik V	Sputnik V		negative	negative
**B01-21/166/MADC**	73	M	Caucasian	Hispanic	Yes	Sputnik V	Sputnik V		30.57	20.01
**B01-21/167/MTPR**	55	F	Caucasian	Hispanic	No	Sputnik V	Sputnik V		negative	N/A
**B01-21/168/YCS**	69	F	Caucasian	Hispanic	Yes	Sputnik V	Sputnik V		21.1	12.74
**B01-21/169/JECL**	57	M	Caucasian	Hispanic	No	Sputnik V	Sputnik V		negative	N/A
**B01-21/170/MLLL**	51	F	Caucasian	Hispanic	No	Sputnik V	Sputnik V		negative	negative
**B01-21/171/FRJ**	58	F	Caucasian	Hispanic	Yes	Sputnik V	Sputnik V	positive	80.31	47.77
**B01-21/172/GMV**	51	F	Caucasian	Hispanic	Yes	Sputnik V	Sputnik V	positive	63.9	39.8
**B01-21/173/MdLMG**	53	F	Caucasian	Hispanic	Yes	Sputnik V	Sputnik V		32.65	18.22
**B01-21/174/BGS**	50	M	Caucasian	Hispanic	Yes	Sputnik V	Sputnik V	positive	200.8	148
**B01-21/175/MdLFMG**	64	F	Caucasian	Hispanic	No	Sputnik V	Sputnik V		negative	negative
**B01-21/176/SFH**	67	M	Caucasian	Hispanic	Yes	Sputnik V	Sputnik V		103.5	29.42
**B01-21/177/CMV**	63	F	Caucasian	Hispanic	Yes	Sputnik V	Sputnik V	positive	70.42	46.47
**B01-21/178/EJFH**	63	F	Caucasian	Hispanic	No	Sputnik V	Sputnik V		negative	N/A
**B01-21/179/FJFH**	61	M	Caucasian	Hispanic	No	Sputnik V	Sputnik V		negative	N/A
**B01-21/180/RBM**	63	M	Caucasian	Hispanic	Yes	Sputnik V	Sputnik V		13.69	5.23
**B01-21/181/MGSN**	52	F	Caucasian	Hispanic	No	Sputnik V	Sputnik V		negative	negative
**B01-21/182/BAG**	64	F	Caucasian	Hispanic	No	Sputnik V	Sputnik V		negative	negative
**B01-21/184/FCM**	65	F	Caucasian	Hispanic	Yes	Sputnik V	Sputnik V	positive	179.6	94.49
**B01-21/185/YJRV**	50	F	Caucasian	Hispanic	No	Sputnik V	Sputnik V		negative	23.62
**B01-21/186/AASP**	71	F	Caucasian	Hispanic	Yes	Sputnik V	Sputnik V	positive	173.3	N/A

**B01-21/248/JTH**	56	F	Caucasian	Hispanic	Yes	Sputnik V	Sputnik V	positive	40.15	54.3
**B01-21/249/SSCZ**	64	F	Caucasian	Hispanic	No	Sputnik V	Sputnik V		negative	N/A
**B01-21/007/GGHT**	30	F	Caucasian	Hispanic	Yes	BNT162b2-BNT	BNT162b2-BNT	positive	139.4	79.01
**B01-21/017/VMDC**	29	F	Caucasian	Hispanic	No	BNT162b2-BNT	BNT162b2-BNT		negative	5.16
**B01-21/038/LLJS**	28	F	Caucasian	Hispanic	Yes	BNT162b2-BNT	BNT162b2-BNT		18.85	11.72
**B01-21/042/MAPR**	70	F	Caucasian	Hispanic	No	BNT162b2-BNT	BNT162b2-BNT		negative	negative
**B01-21/045/ECAC**	79	F	Caucasian	Hispanic	No	BNT162b2-BNT	BNT162b2-BNT		negative	negative
**B01-21/065/GAOL**	65	F	Caucasian	Hispanic	Yes	BNT162b2-BNT	BNT162b2-BNT		negative	negative
**B01-21/069/AAGH**	27	M	Caucasian	Hispanic	No	BNT162b2-BNT	BNT162b2-BNT		negative	negative
**B01-21/077/LCU**	43	M	Caucasian	Hispanic	No	BNT162b2-BNT	BNT162b2-BNT		negative	negative
**B01-21/079/CJHE**	27	F	Caucasian	Hispanic	No	BNT162b2-BNT	BNT162b2-BNT		negative	negative
**B01-21/081/AKFE**	24	F	Caucasian	Hispanic	Yes	BNT162b2-BNT	BNT162b2-BNT		134.7	69.21
**B01-21/082/AMTX**	25	F	Caucasian	Hispanic	No	BNT162b2-BNT	BNT162b2-BNT		negative	negative
**B01-21/086/CKVC**	26	F	Caucasian	Hispanic	No	BNT162b2-BNT	BNT162b2-BNT		negative	negative
**B01-21/087/VSHG**	24	F	Caucasian	Hispanic	No	BNT162b2-BNT	BNT162b2-BNT		negative	11.78
**B01-21/091/AGP**	32	F	Caucasian	Hispanic	Yes	BNT162b2-BNT	BNT162b2-BNT	positive	102.8	186.1
**B01-21/092/EEC**	58	M	Caucasian	Hispanic	No	BNT162b2-BNT	BNT162b2-BNT		negative	negative
**B01-21/097/RMAG**	67	F	Caucasian	Hispanic	Yes	BNT162b2-BNT	BNT162b2-BNT	positive	26	22.3
**B01-21/098/ROA**	39	F	Caucasian	Hispanic	No	BNT162b2-BNT	BNT162b2-BNT		negative	negative
**B01-21/100/SVB**	65	F	Caucasian	Hispanic	No	BNT162b2-BNT	BNT162b2-BNT		negative	negative
**B01-21/101/MGCV**	61	F	Caucasian	Hispanic	No	BNT162b2-BNT	BNT162b2-BNT		negative	negative
**B01-21/103/RSFL**	60	F	Caucasian	Hispanic	No	BNT162b2-BNT	BNT162b2-BNT		negative	negative
**B01-21/108/MESS**	69	F	Caucasian	Hispanic	No	BNT162b2-BNT	BNT162b2-BNT		negative	negative
**B01-21/109/MACC**	47	M	Caucasian	Hispanic	Yes	BNT162b2-BNT	BNT162b2-BNT		3.22	3.13
**B01-21/110/RAN**	28	M	Caucasian	Hispanic	Yes	BNT162b2-BNT	BNT162b2-BNT	positive	98.41	67.17
**B01-21/111/RHO**	50	M	Caucasian	Hispanic	No	BNT162b2-BNT	BNT162b2-BNT		negative	negative
**B01-21/156/MdLN**	58	F	Caucasian	Hispanic	No	BNT162b2-BNT	BNT162b2-BNT		negative	negative
**B01-21/242/AIG**	65	F	Caucasian	Hispanic	Yes	BNT162b2-BNT	BNT162b2-BNT	positive	168.2	77.67
**B01-21/247/BJGO**	45	F	Caucasian	Hispanic	Yes	BNT162b2-BNT	BNT162b2-BNT	positive	2.97	1.34
**B01-21/284/VFELdMGSS**	63	F	Caucasian	Hispanic	Yes	BNT162b2-BNT	BNT162b2-BNT		2.57	negative

Anti-N antibodies were measured on the day of blood donation by ELISA and AUC values are shown.
